# Oral microbiome brain axis and cognitive performance in older adults

**DOI:** 10.1038/s44400-025-00004-4

**Published:** 2025-04-07

**Authors:** Darbaz Adnan, Phillip A. Engen, Michelle Villanueva, Shohreh Raeisi, Vivian Ramirez, Ankur Naqib, Stefan J. Green, Faraz Bishehsari, Lisa L. Barnes, Ali Keshavarzian, Klodian Dhana, Robin M. Voigt

**Affiliations:** 1 Rush Center for Integrated Microbiome and Chronobiology Research, Rush Medical College, Rush University Medical Center, Chicago, IL, USA.; 2 Genomics and Microbiome Core Facility, Rush University Medical Center, Chicago, IL, USA.; 3 Department of Internal Medicine, Rush University Medical Center, Chicago, IL, USA.; 4 Gastroenterology Research Center, Division of Gastroenterology, Hepatology & Nutrition, Department of Internal Medicine, University of Texas Health Science Center at Houston, Houston, TX, USA.; 5 Rush Alzheimer’s Disease Center, Rush University Medical Center, Chicago, IL, USA.; 6 Department of Anatomy and Cell Biology, Rush University Medical Center, Chicago, IL, USA.; 7 Rush Institute for Healthy Aging, Rush University Medical Center, Chicago, IL, USA.

## Abstract

The human oral microbiota is a community of microorganisms that reside in the oral cavity, including lingual, buccal, and saliva, each niche with a distinct microbial composition. Alterations in oral microbiota have been associated with an increased risk of Alzheimer’s disease (AD). This study used data from 143 older adults in the MIND trial to evaluate the association between oral microbiome and cognitive function. Oral niche-specific differences (saliva, buccal, and lingual), as well as the microbiome composition differences (α and β diversity), were associated with cognitive function. A lower abundance of *Gemella* and a higher abundance of anaerobic pro-inflammatory bacteria (e.g., *Parvimonas*, *Treponema*, *Dialister*) were linked to a lower Cognitive Z Score. *Porphyromonas*, previously linked to AD, was not associated with cognition. The outcomes suggest that oral microbiota may be a biomarker for cognitive function. Further research is required to assess whether oral microbiota-directed strategies can positively impact cognitive decline.

Alzheimer’s disease (AD) is a progressive neurodegenerative disease characterized by progressive loss of cognitive function^1^. Genetics plays a role in AD pathogenesis, but environmental and lifestyle factors are also thought to be important. One important lifestyle factor is oral health, which refers to the health of the mouth, teeth, and gums.

Periodontal disease, also known as gum disease, is a term used to describe the inflammation that affects the tissues responsible for anchoring teeth, including the gingiva, supporting connective tissue, and alveolar bone^2^. This condition is believed to be caused by a pro-inflammatory oral microbiome (i.e., dental plaque and bacterial biofilm) that accumulates on teeth and gums, leading to damage of the connective tissue and alveolar bone, eventually resulting in tooth loss^3^. Lifestyle factors, such as diet, smoking, and oral hygiene, influence the oral microbiome and contribute to the development and progression of periodontal disease^4^.

Studies have revealed a compelling link between oral hygiene and periodontal disease in AD^5–7^. For example, individuals with no teeth or few teeth (signs of severe periodontal disease) are more likely to develop dementia and have a faster progression of dementia than individuals without tooth loss^8,9^. Additionally, individuals with AD have increased levels of antibodies against periodontal pathogens in their blood (e.g., *Porphyromonas gingivalis*) compared with controls without dementia^10–12^. Finally, individuals with AD exhibit a distinct oral bacterial composition compared with those without AD^13–19^. Bacterial species such as *P. gingivalis*, *Treponema denticola*, *Prevotella intermedia*, and *Parvimonas micra* are more abundant in individuals with AD than in cognitively normal individuals^20^. These studies evaluated individuals with a clinical diagnosis of AD; however, AD takes decades to develop, and the extent of microbial changes prior to AD diagnosis (e.g., during mild cognitive impairment (MCI)) is less studied. A recent report found that pro-inflammatory changes in the salivary microbiome are a feature among patients with MCI compared to cognitively normal controls^21^, but this phenomenon has not been examined in other populations or in other oral microbiome niches (e.g., buccal and lingual).

This study evaluated the relationships between the oral microbial community niches and cognitive function in older adults. The salivary, buccal, and lingual microbial communities were examined in individuals with a self-reported family history of AD, but who did not have a clinical diagnosis of AD. The results demonstrated significant differences in oral microbiota based on cognitive function; individuals with lower cognition had an increased abundance of pro-inflammatory microbes and reduced abundances of microbes thought to be protective compared to individuals with higher cognition. These results demonstrate that the oral microbiota is associated with cognitive function; future studies are needed to determine whether the oral microbiota may function as biomarkers to predict AD.

## Results

In this cross-sectional study, 143 older adults provided buccal, lingual, and saliva samples. Among the 143 participants, 103 (72%) were females with a mean age of 73 years (68.1–86.5). The demographic and biomedical data are shown in Table 1 and Supplementary Table 2, respectively.

### Oral niche microbiota profiles

**Fig. 1 |:**
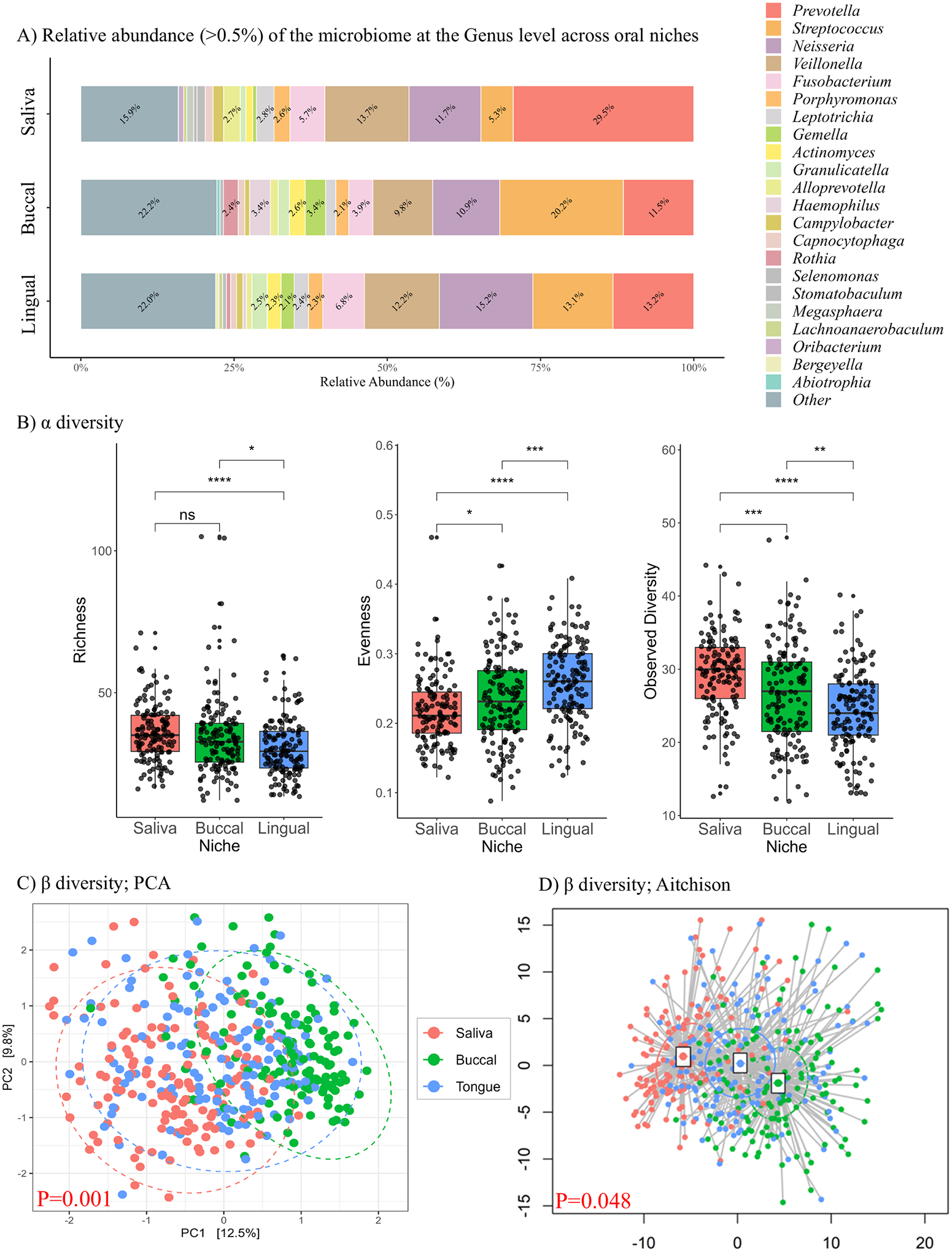
The distinction of microbiome across oral niches. **A** A stacked bar plot showing taxa at the genus level with an abundance above 0.5% for each oral niche. *Prevotella* was most abundant in saliva, *Streptococcus* in buccal, and *Neisseria* in the lingual niche. **B** Box plots showing significant differences in α-diversity indices across oral niches. Wilcoxon rank test: *p*-values: ns = not significant, *p < 0.05, **p < 0.01, ***p < 0.001, ***p < 0.0001. **C**, **D** Plots show significant variation in microbiome composition (β diversity) between different oral niches.

Analysis revealed the presence of similar taxa among all niches (salivary, buccal, and lingual), including *Prevotella*, *Streptococcus*, *Neisseria*, and *Veillonella*. *Prevotella* emerged as the predominant genus in saliva, *Streptococcus* in the buccal, and *Neisseria* in the lingual, demonstrating distinctive patterns of taxonomic abundances across different oral niches (Fig. 1A). Next, α-diversity indices were computed at the genus level across the different oral niches for richness (Chao1), evenness (Simpson’s Evenness), and bacterial diversity (observed features). Each oral niche displayed a distinct α-diversity profile. The lingual niche exhibited significantly lower richness and observed diversity compared to the other oral niches, along with the highest evenness. In contrast, the saliva niche showed greater richness and observed features but had the lowest evenness. (Fig. 1B). Beta-diversity (β diversity) analysis at the genus level, exhibited significant differences across oral niches (Fig. 1C, PERMANOVA test: *F* = 16.9, *R*^2^ = 0.074, *p* = 0.001, Fig. 1D: ordination analysis *p* = 0.048). Due to the microbial community structure differences between the oral niches, each oral sampling site was assessed independently in subsequent analyses.

### Relationship between oral microbiota and cognition

**Fig. 2 |:**
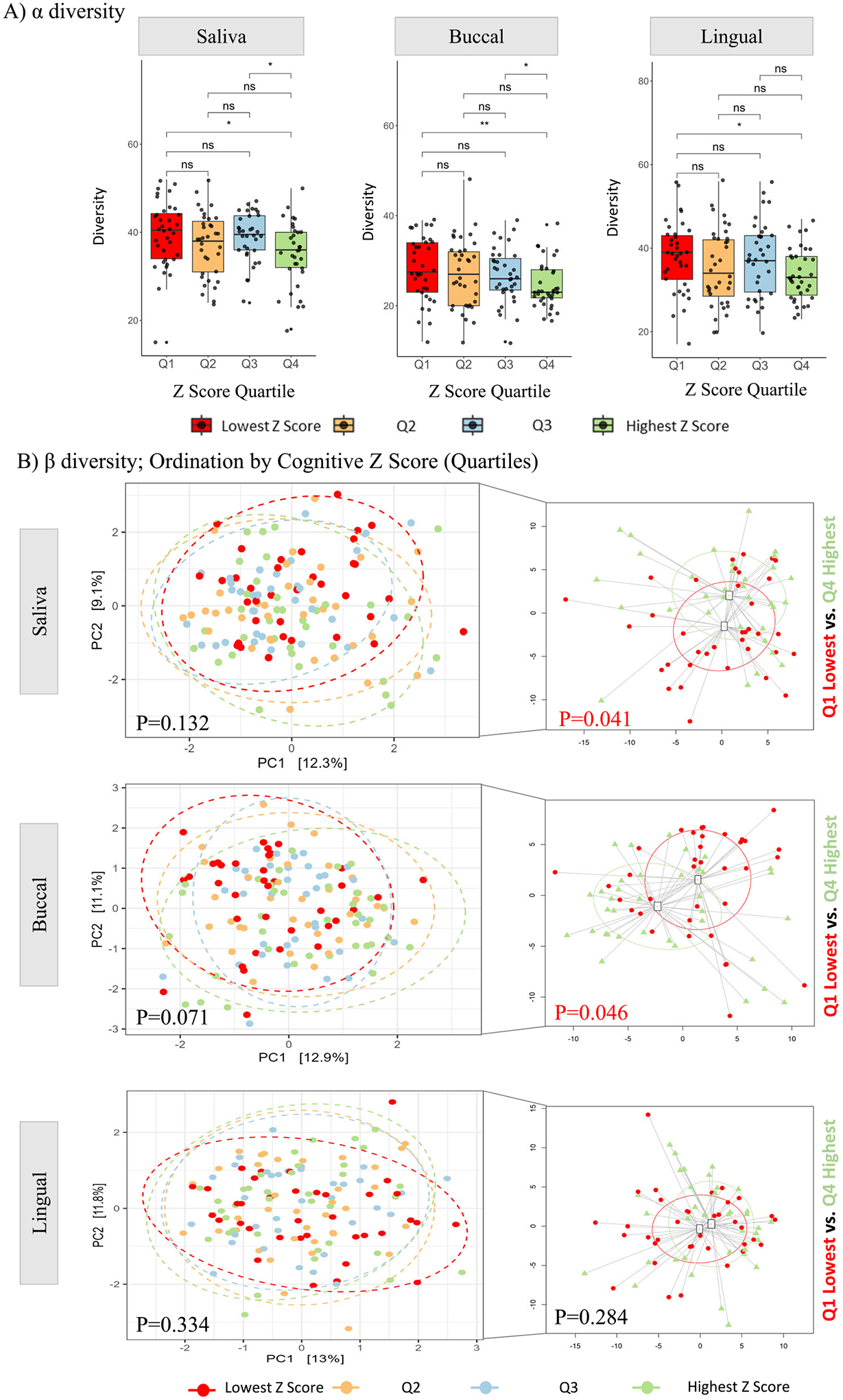
Microbiome diversity by cognitive Z score. **A** The α-diversity index of the oral microbiome community by cognitive Z Score quartile is shown for saliva, buccal, and lingual niches. α-diversity was significantly different between the Q1 (lowest cognitive Z scores) and Q4 (highest cognitive Z Scores) quartiles in all oral niches (*p* < 0.05). Wilcoxon rank test: *p*-values: ns = not significant, *p < 0.05, **p < 0.01. **B** Variations in oral microbiome composition (β diversity) across cognitive Z Score quartiles were assessed using the Bray-Curtis dissimilarity matrix and tested with PERMANOVA. Significant variations in β-diversity were observed between the Q1 and Q4 Cognitive Z Score quartiles in the saliva and buccal niches. Q = quartile, Q1 = lowest cognitive Z Score, Q4 = highest cognitive Z Score.

**Fig. 3 |:**
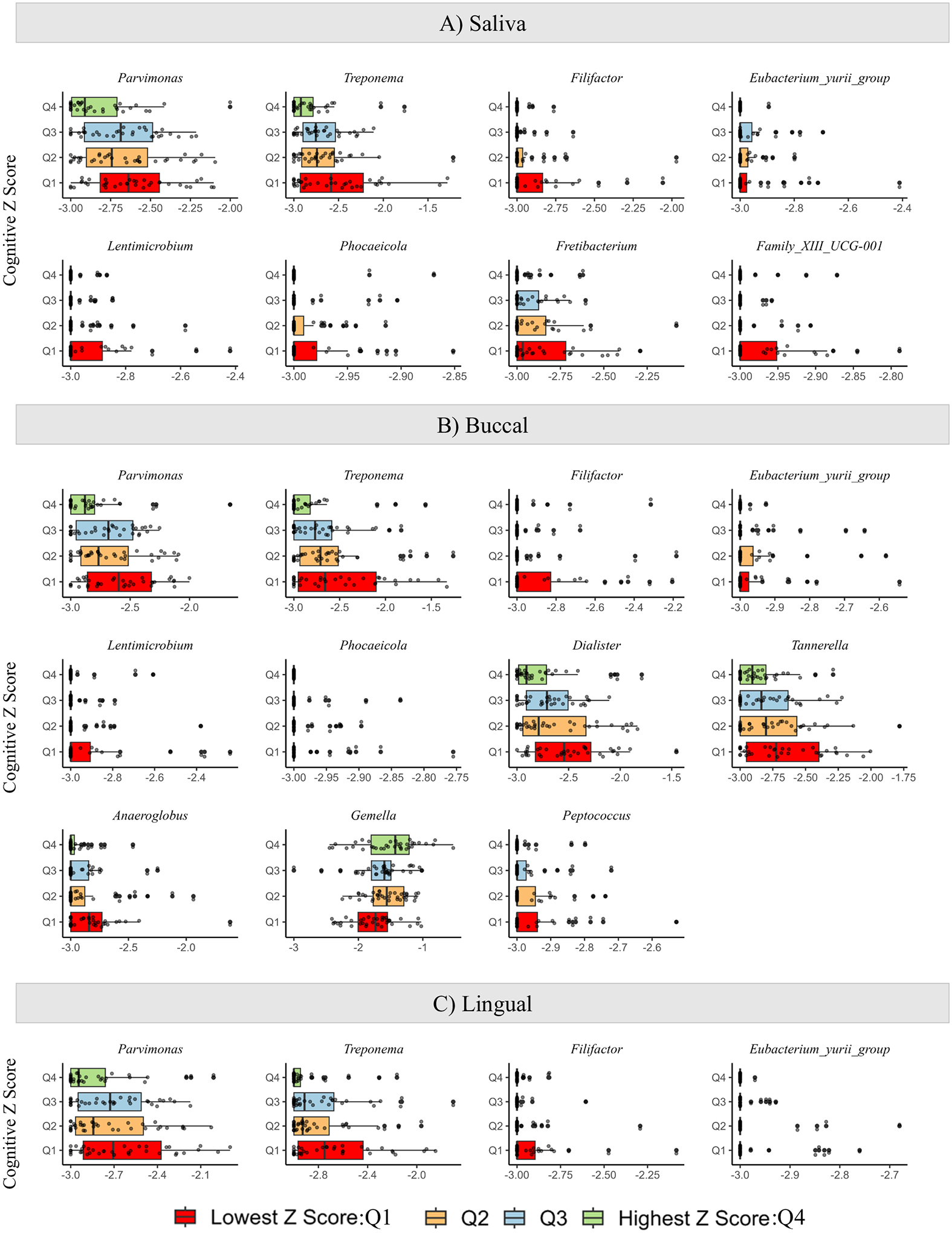
Differential abundance by cognitive Z score. Box plots display the significantly differentially abundant taxa at the genus level across cognitive Z Score quartiles for each oral niche: **A** saliva, **B** buccal, and **C** lingual (all *p* < 0.05, *q* < 0.25). Eleven taxa showed statistically significant differences in abundance across all three niches (displayed on the *Y*-axis). Among these, four taxa—*Parvimonas*, *Treponema*, *Filifactor*, and *Eubacterium_yurii_group*—were consistently significantly different across all oral niches. Analysis was conducted using maaslin2 utilizing a multivariable linear model by cognitive Z Score, adjusting for MIND diet score, smoking history, and sex. The criteria for significance included a *p*-value < 0.05 and *q*-value < 0.25. Q = quartile, Q1 = lowest cognitive Z Score, Q4 = highest cognitive Z score.

Initially, α- and β-diversity at the genus level in oral microbial composition were assessed among participants across different cognitive Z Score quartiles (Q1: Lowest Z Score, Q2, Q3, Q4: Highest Z Score). The Wilcoxon rank test revealed participants with the lowest cognitive Z Score (Q1) exhibited a statistically significant higher observed diversity in each oral niche compared to participants with the highest cognitive Z Score (Q4) (Fig. 2A; Saliva *p* = 0.016, buccal *p* = 0.009, lingual *p* = 0.016). Participants with the lowest cognitive Z Score (Q1) exhibited higher richness (Chao1) and the lowest evenness than participants with the highest cognitive Z Score (Q4) across all oral niches (Supplementary Table 3). To better understand the microbial compositional differences in the oral microbiome based on the cognitive Z score, a PERMANOVA analysis was conducted, at the genus level, and an ordination plot based on cognition quartiles was generated (Fig. 2B). Analysis comparing all four cognitive quartiles revealed no significant differences. However, significant differences were observed between those with the lowest cognitive Z Score (Q1) and those with the highest cognitive Z Score (Q4) for saliva and buccal niches (PERMANOVA: saliva *p* = 0.041, buccal *p* = 0.046, lingual *p* = 0.284, Fig. 2B). The results from both α- and β-diversity analyses indicated significant differences in microbiota compositions based on cognitive status. These microbiota findings remained consistent even after accounting for diet, sex, education, use of antibiotics, and smoking (factors known to influence the oral microbiota, Supplementary Fig. 1A–I)^4,22,23^. In fact, the Cognitive Z Score had a more robust impact on microbial diversity than the other factors examined (saliva: *R*^2^ = 0.216, buccal: *R*^2^ = 0.156, lingual: *R*^2^ = 0.227) (Supplementary Fig. 1G–I). Due to the significant effects of diet, smoking history, and sex on microbial diversity, subsequent analyses were corrected for these covariates (details of diet, smoking history, and sex are shown in Table 1). The level of education of the participants did not affect the microbial diversity across all three oral niches (Supplementary Fig. 1G–I), and it was also not significantly different between the Cognitive Z Score quartiles (Table 1). Only two participants, one with a cognitive Z Score falling within Q2, and the other within Q3, used oral antibiotics within one month of sample collection (Supplementary Fig. 1G–I). Therefore, the subsequent analysis did not adjust for education or antibiotic use. Supplementary Fig. 2A–C shows the relative abundance of taxa above 0.5% across the three oral niches. Differential abundance analysis was conducted to identify taxa associated with cognitive Z Score. Univariate analysis (Taxa ~ Cognitive Z Score) identified 28 unique taxa that were significantly different in all oral niches combined (Supplementary Table 4). Next, genus enrichment analysis was conducted accounting for confounding covariates (Taxa ~ Cognitive Z Score + Diet + Smoking + Sex) to assess differences in taxa between cognitive Z Score quartiles. A total of eight taxa in saliva, 11 taxa in buccal, and four taxa in lingual niches exhibited significant differences in abundance across the Cognitive Z Scores (Fig. 3, Supplementary Fig. 3, Supplementary Table 5). *Parvimonas*, *Treponema*, *Filifactor*, and *Eubacterium yurii* were consistently differentially abundant across groups in each niche. *Lentimicrobium* and *Phocaeicola* were differentially abundant in the saliva and buccal niches, while *Fretibacterium* and *Family_XII_UCG-001* were only found to be differentially abundant in the salivary niche. Additionally, *Gemella*, *Dialister*, *Tannerella*, and *Anaeroglobus* were differentially abundant in the buccal niche. All bacteria mentioned above were found to be more abundant in individuals with a lower cognitive Z Score compared to those with a higher cognitive Z Score, except for *Gemella*, which was notably less abundant in those with a lower Cognitive Z Score. Collectively, these findings indicate that each oral niche has a specific microbiota composition that differs based on the cognitive Z Score (Fig. 3).

**Fig. 4 |:**
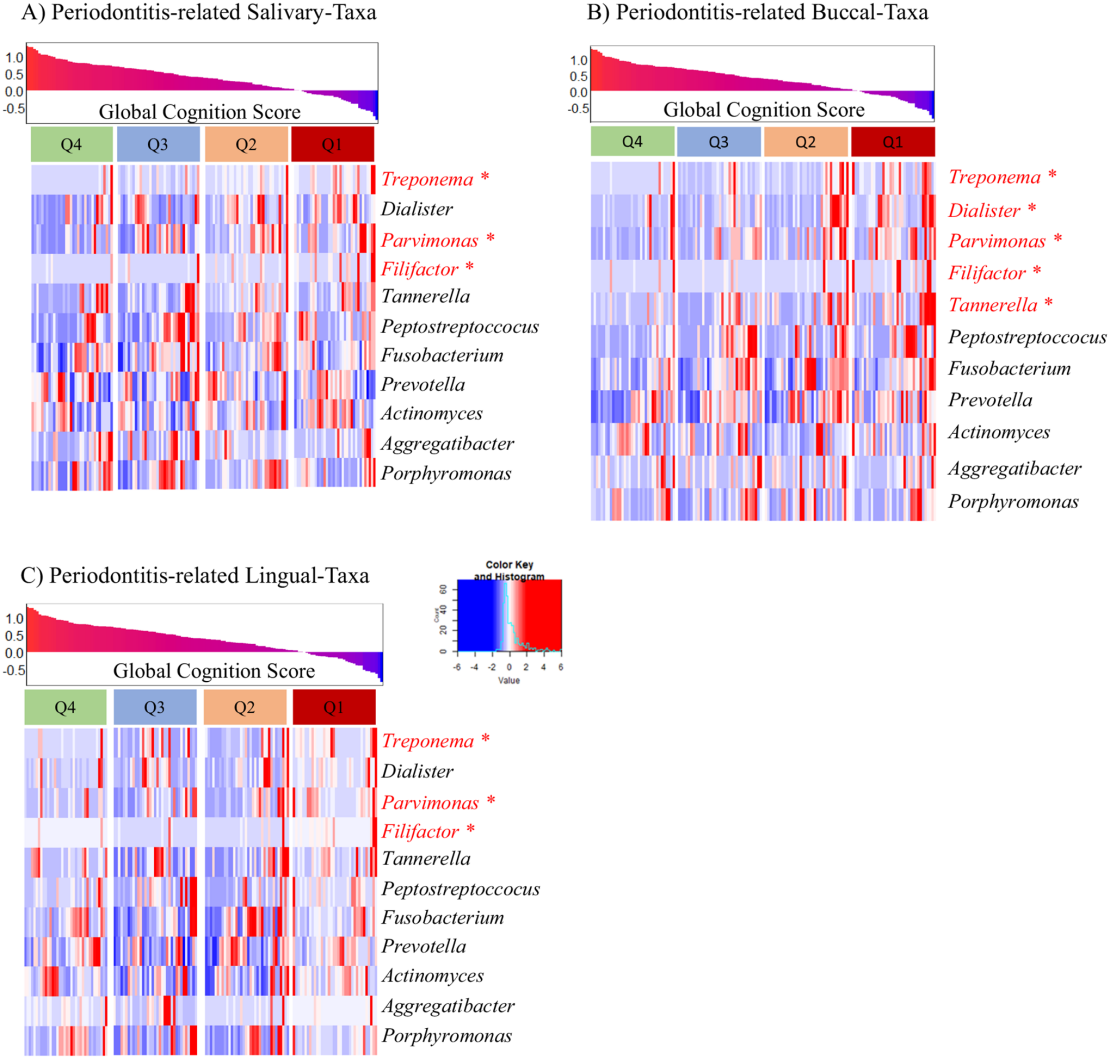
Periodontitis-related taxa by cognitive Z score. Heatmaps show the relative abundance of 11 periodontitis-related taxa across cognitive Z Score quartiles for each oral niche: saliva, buccal, and lingual. The cognitive Z Score quartiles are indicated in the top panel of each heatmap. The significant taxa are indicated by red asterisks (*). **A** Saliva: Analysis revealed that three periodontitis-related taxa (*Treponema*, *Parvimonas*, and *Filifactor*) were statistically significant (*p* < 0.05, *q* < 0.25). **B** Buccal: Analysis revealed five periodontitis-related taxa (*Treponema*, *Dialister*, *Parvimonas*, *Filifactor*, and *Tannerella*) were statistically significant (*p* < 0.05, *q* < 0.25). **C** Lingual: Analysis revealed that three periodontitis-related taxa (*Treponema*, *Parvimonas*, and *Filifactor*) were statistically significant (*p* < 0.05, *q* < 0.25). Analysis was conducted using maaslin2 utilizing a multivariable linear model by cognitive Z Score adjusting for MIND diet score, smoking history, and sex. Q = quartile, Q1 = lowest cognitive Z Score, Q4 = highest cognitive Z Score.

Next, we a priori selected 11 taxa known to be associated with periodontitis (*Treponema*, *Dialister*, *Parvimonas*, *Filifactor*, *Tannerella*, *Peptostreptococcus*, *Fusobacterium*, *Prevotella*, *Actinomyces*, *Aggregatibacter*, and *Porphyromonas*) and evaluated their relationship with cognitive Z Score. Among the taxa, three were enriched in the Q1 cognitive Z Score group in all three niches (saliva, buccal, and lingual), including *Treponema*, *Parvimonas*, and *Filifactor*. Two additional taxa were enriched in Q1 in the buccal niche, including *Dialister* and *Tannerella*. These results indicated that some periodontitis-related taxa were enriched in people with a lower cognitive Z Score, and among the oral niches, the buccal region had the greatest number of associations between periodontitis and cognition (Fig. 4). Additionally, there was no relationship between the APOE genotype and oral microbiota (data not shown). Univariate analysis of all tested biomarkers by Cognitive Z Score is shown in Supplementary Table 6.

### Relationship between oral microbiota and inflammation, cholesterol panel, biomarkers of AD

**Fig. 5 |:**
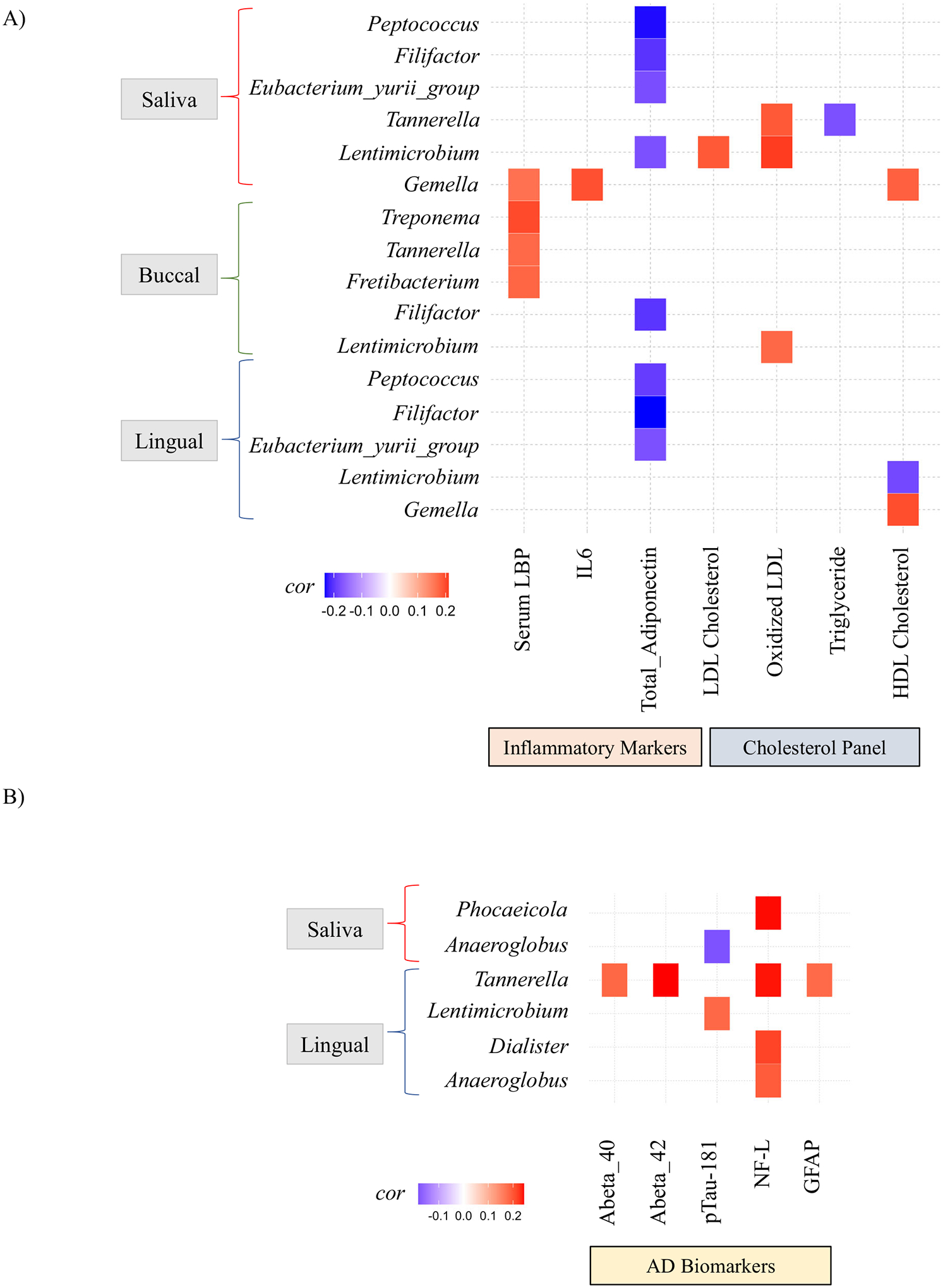
Microbiome associations with plasma AD markers, inflammation. Correlation analysis showing associations of the oral microbiome from three niches (saliva, buccal, and lingual) with systemic inflammatory markers, plasma cholesterol, plasma Alzheimer’s disease (AD) markers. **A** Spearman’s correlation coefficients are shown for significant taxa, inflammatory markers, and cholesterol levels. **B** Spearman correlation coefficients are shown for statistically significant taxa and plasma AD markers. All correlations were statistically significant at *p* < 0.05 and *q* < 0.25.

We evaluated the relationship between the oral microbiota and systemic inflammation (i.e., plasma markers of inflammation) using Spearman’s correlation analysis, which showed a significant correlation (*p* < 0.05) between oral microbes and systemic inflammatory markers. Salivary *Gemella* and buccal *Fretibacterium*, *Tannerella*, and *Treponema* were positively correlated with LBP, an important component of the immune system’s response to Gram-negative bacteria that is elevated in AD^24^. Salivary *Gemella* was positively correlated with IL-6. Additionally, salivary *Peptococcus*, *Lentimicrobium*, *Eubacterium_yurii_group*, and *Filifactor*, along with buccal *Filifactor*, and lingual *Filifactor*, *Peptococcus. Eubacterium_yurii_group* negatively correlated with adiponectin levels, which is typically anti-inflammatory and lower in patients with AD than in cognitively normal individuals^25,26^ (Fig. 5A). Overall, bacterial taxa in the various oral niches are associated with inflammation.

We then investigated the relationship between the oral microbiota and cholesterol levels. Salivary *Tannerella* was positively correlated with oxidized LDL and negatively correlated with triglycerides. Salivary *Lentimicrobium* positively correlated with both LDL and oxidized LDL levels. Salivary *Gemella* exhibited a positive correlation with HDL. Lingual *Lentimicrobium* showed a negative correlation with HDL, whereas lingual *Gemella* was positively correlated with HDL (Fig. 5A).

Next, we explored the association between the oral microbiota and plasma biomarkers of AD. The analysis revealed that lingual *Tannerella* positively correlated with plasma Aβ 40 and Aβ 42. The neurodegeneration marker NF-L positively correlated with salivary *Phocaeicola*, lingual *Anaeroglobus*, *Tannerella*, and *Dialister*. GFAP (a marker of neuroinflammation that is elevated in AD^27^) positively correlated with lingual *Tannerella* (Fig. 5B). Univariate analysis of the AD biomarkers based on Cognitive Z Score revealed a trend for increased presence of all tested blood AD biomarkers (Aβ 40, Aβ 42, pTau, NF-L, GFAP) in Q1 relative to other quartiles; however, this was not statistically significant (*p* > 0.05, Supplementary Table 6). A univariate differential abundance analysis was conducted to evaluate the relationship between the oral microbiota and plasma biomarkers of AD, regardless of Cognitive Z Score (Supplementary Table 7). Salivary *Rothia* positively correlated with plasma Aβ 40, Aβ 42, NF-L, pTau, while lingual *Rothia* positively associated with Aβ 40, Aβ 42, NF-L. Furthermore, salivary *Alloprevotella*, *Lachnoanaerobaculum*, *Megasphaera*, *Mogibacterium*, *Oribacterium* and *Stomatobaculum* were negatively associated with NF-L. Salivary *Gemella* and *Streptococcus* were positively associated with plasma NF-L. Additionally, lingual *Granulicatella* and *Stomatobaculum* negatively associated with Aβ 42 and lingual *Stomatobaculum* negatively associated with Aβ 40. Finally, lingual *Lentimicrobium* and *Abiotrophia* positively associated with Aβ 42. These results suggest that *Rothia* (salivary and lingual) has the strongest correlation with blood biomarkers of AD, and the oral microbiota is associated with certain pathological markers of AD, even in individuals who do not have a clinical diagnosis of AD.

### Relationship between oral microbiota and brain structure (MRI)

Associations between the oral microbiota and MRI assessment of brain structure/volume, including hippocampal volume, gray and white matter (WM) volume, and white matter hyperintensities, were evaluated. There were no statistically significant correlations between oral microbiota and brain structure assessed through an MRI.

### Relationship between oral microbiota and dietary components

Given the nature of this study as an ancillary study of the MIND trial, we analyzed oral microbiota diversity and differential abundance by group assignment (i.e., MIND diet intervention or Control diet). Analysis revealed no significant difference in α- or β-diversity between MIND diet intervention or Control diet in any oral niche (Supplementary Fig. 4A, B). Univariate and multivariate differential abundance analysis (Taxa ~ Group Assignment + Cognitive Z Score + Smoking + Sex) revealed that two genera, *Dialister* and *Selenomonas*, were differentially abundant in the buccal niche based on group assignment (i.e., Control vs MIND) (Supplementary Fig. 4C).

**Fig. 6 |:**
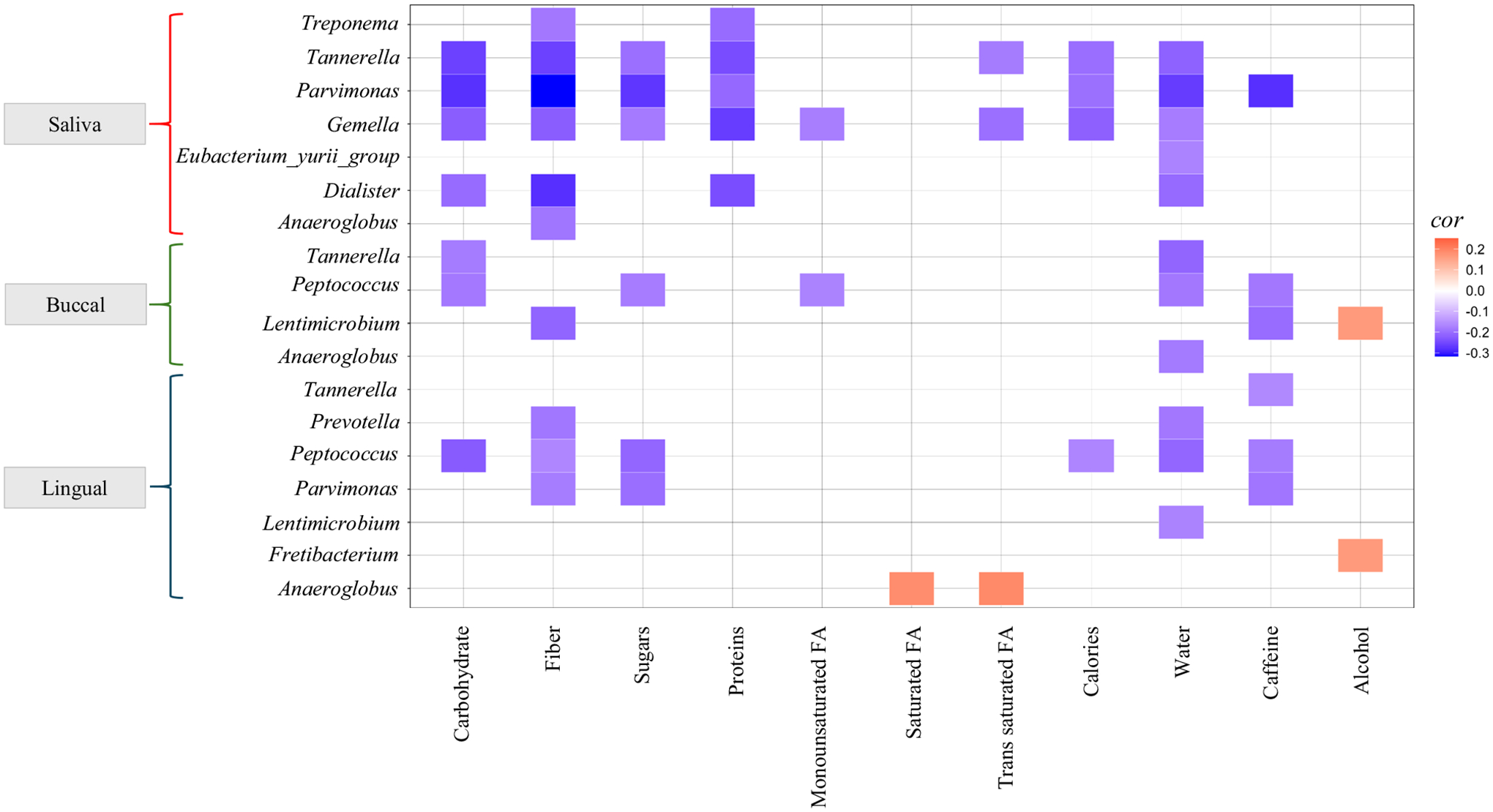
Oral microbiome associations with dietary components. The correlation analysis (Spearman’s correlation) demonstrates statistically significant associations between the oral microbiome from three niches (saliva, buccal, and lingual) and dietary components. All correlations were statistically significant at *p* < 0.05 and *q* < 0.25.

Since redundancy analysis revealed that the MIND diet score (regardless of group assignment) accounted for 2–5% of oral microbial diversity (Supplementary Fig. 1), we further explored the variation in microbiota dissimilarity based on MIND diet score. Analysis revealed that α- and β-diversity were not significantly different across the MIND diet score quartiles (Supplementary Fig. 5A, B), and no taxa were differentially abundant. To assess whether various dietary components correlated with the abundance of cognition-associated taxa (identified in Fig. 3), a Spearman correlation analysis was conducted for each oral niche. Analysis revealed that bacterial taxa enriched in participants with lower cognitive Z Score correlated negatively with the consumption of carbohydrates, fiber, sugars, proteins, and consumption of water and caffeine, however, positively correlated with fat and consumption of alcohol (Fig. 6). The negative correlation between many microbes with diet and water consumption is interesting, as reports suggest that people with dementia consume less food and water^24,26^.

## Discussion

Emerging evidence demonstrates that the oral microbiome is distinct in individuals with AD and mild cognitive impairment (MCI) compared with cognitively normal individuals^28^. In this study, multiple oral microbiota niches (saliva, buccal, and lingual) were examined in well-phenotyped participants of advanced age. Alpha-diversity indices were highest in participants with the lowest cognitive Z Scores (Q1) compared to participants with the highest cognitive Z Scores (Q4) in all oral niches examined (Fig. 2A), and beta-diversity between the highest and lowest Z Scores (Q1 vs. Q4) was significantly different in the salivary and buccal niches (Fig. 2B). Participants with the lowest cognitive Z Scores (Q1) had microbial communities with increased relative abundances in putative pro-inflammatory bacteria, with a depleted abundance in genus *Gemella*, which has been associated with optimal cognitive performance^21^ (Fig. 3). Additionally, bacteria associated with periodontitis were enriched in participants with the lowest cognitive Z Score (Q1) (Fig. 4). Finally, there was a significant but weak correlation between oral taxa that were differentially abundant based on cognition, systemic inflammation, and plasma markers of AD. The literature suggests that MCI is associated with an altered oral microbiome^21,29^, and the results of this study suggest that multiple oral microbiota niches should be evaluated to understand the potential importance of the oral microbiome to cognitive function and AD.

Recent studies demonstrate that the oral microbiome varies depending on cognitive function^13–19^. One study reports an increased abundance of gram-negative bacteria in the salivary niche of participants with AD and MCI compared to cognitively normal individuals^29^, while another study reported a decreased abundance in pro-inflammatory bacteria^30^. Differences in outcomes likely reflect different study populations, diagnostic criteria for AD and MCI, and sample collection (including niche) and analysis approaches. However, multiple studies have reported an increase in microbial diversity in individuals with AD and MCI compared to cognitively normal individuals^16,28–30^ (although other studies have reported no differences^31,32^). This is similar to what was observed in the current study, wherein those with the lowest cognitive Z Score (Q1) had higher microbial diversity than individuals with the highest cognitive Z Score (Q4), which was true in the buccal saliva and lingual niches (Fig. 2A).

The specific bacteria identified as relevant for AD/MCI in previous studies are divergent, indicating that additional evaluations are needed with attention to specific microbiome niches. *P. gingivalis* is one taxon that has been associated with AD^33^, but this taxon did not emerge as important in the current study. The genus *Gemella* was depleted in the buccal niche in those with the lowest cognitive Z Score (Q1, Fig. 3). *Gemella* has been proposed as a bacteria that may protect cognitive function^21^. For example, a recent co-culture experiment demonstrated that the supernatant from *Gemella* spp. inhibited the growth of *P. gingivalis*^34^, a major microbe that causes periodontitis and has been found to be associated with AD^33^. *Gemella* is also positively correlated with HDL, a type of cholesterol associated with AD^35,36^. *Parvimonas* and *Treponema* were enriched in those with the lowest cognitive Z Scores (Q1) compared to individuals with higher cognitive Z Scores (Q4) (Figs. 3, 4). In a previous report, 14 out of 16 deceased individuals with AD had *Treponema* in brain tissue and the trigeminal nerve, suggesting that *Treponema* may migrate from the mouth through the trigeminal nerve to the brain^37^. Animal studies have shown that oral administration of *Treponema denticola* to C57BL/6 mice can cause accumulation of amyloid β 42 and induce neuronal damage in the hippocampus^38,39^. *Parvimonas* has been identified as a potential causative factor in the development and progression of periodontitis^40^; however, the impact of *Parvimonas* on cognitive decline has yet to be explored. Further oral microbiota studies should explore the enrichment of *Treponema* and *Parvimonas* and loss of *Gemella* as potential preclinical biomarkers of MCI and AD.

The gut microbiota can influence cholesterol levels^41–43^. Genetics, diet, and lifestyle habits influence host cholesterol levels, but the gut microbiome influences the absorption of cholesterol in the intestine^42,44^. LDL and oxidized LDL are indirectly associated with an increased risk of AD, while HDL is associated with a decreased risk^45^. The contribution of the oral microbiome to cholesterol levels is unclear, but the weak associations observed between the oral microbiota and cholesterol levels may reflect dietary habits.

This study identified a significant but weak association between pro-inflammatory bacteria and markers of inflammation and AD biomarkers (Aβ 40, Aβ 42, pTau-181, NF-L, GFAP, Adiponectin, Fig. 5). These findings suggest a potential connection between periodontitis-associated microorganisms, inflammation, and AD biomarkers. While these results may support the hypothesis that microbes serve as causal agents influencing cognitive function, perhaps via inflammation, the cross-sectional nature of this study limits the ability to establish causation. However, salivary microbiome composition is reported to differ between individuals with MCI and cognitively normal individuals^21^, which is supported by the observations in the current study, suggesting that changes in the microbiota may precede AD diagnosis. Furthermore, examination of the relationship between microbiome abundance and blood AD biomarkers, independent of cognitive status, revealed that the higher levels of these blood-based AD biomarkers significantly changed the abundance of many microbes. Especially the genus *Rothia*, which was enriched in the salivary and lingual niches of subjects who had higher levels of Aβ 40, Aβ 42, NF-L, and pTau. These results suggest that some microbes might reflect AD pathology or, more broadly, cognitive decline. More research is required to better understand the relationship between the oral microbiome and AD pathogenesis using AT(N) categorization^46^.

The oral microbiome may influence cognitive function and AD risk via several mechanisms. First, direct invasion of the brain by various oral pathogens (e.g., *Treponema*) can trigger inflammation and accumulation of amyloid, which is an anti-microbial peptide^37,47–51^. Indirectly, the oral microbiome can influence systemic inflammation, which can impact neuroinflammation as well as influence the blood-brain barrier^52^. Additionally, the oral microbiome influences cardio-metabolic health, which is important since cardiovascular disorders (e.g., diabetes) can impact the risk of AD^53^. Lastly, the oral microbiota can impact the gut microbiota and subsequent effects on the gut-brain axis (e.g., metabolites). Studies conducted in animals show that microbiota (particularly in the gut) can contribute to the development of pathological proteins in the brain associated with AD (i.e., amyloid β, tau)^54,55^. Research demonstrates a strong correlation between oral and gut microbiota in both health and diseases^56^. Despite the differences in microbial communities, both oral and gut microbiota share many common microbes. Microbes may translocate from oral to gut and induce dysbiosis, including in patients with periodontitis^57^; nevertheless, the potential impact and relationship of oral dysbiosis on the gut microbiota, and vice versa, in the context of dementia remains unexamined. Additional research is necessary to clarify the comprehensive link between oral and gut microbiota on systemic markers in the context of dementia.

There are some limitations that should be considered when interpreting the results of this study. The role of oral hygiene (i.e., use of floss, mouthwash, types of toothpaste through oral/health questionnaires and dental examinations) and other lifestyle factors in mediating the relationship between the oral microbiota and cognitive function were not explored in this study. The study population was predominantly white (*n* = 121, 84.6%); therefore, the interpretations of this study are limited to this demographic group, as the results cannot be generalized to a broader, diverse population. Moreover, non-whites were enriched in the Cognitive Z Score Q1, and race may have played a role in the between-group differences and future evaluations must carefully address race. Additionally, we employed short-read 16S rRNA gene amplicon sequencing instead of full-length 16S rRNA gene sequencing or shotgun metagenomics, which limited our taxonomic resolution to the genus level rather than the species level. It’s important to note that the high amount of host DNA in our samples decreases the efficiency of shotgun metagenome sequencing. Despite the significant associations (*p* < 0.05 and *q* < 0.25), the correlation coefficients were quite small (i.e., <0.30), indicating that additional factors besides the oral microbiome are influencing levels of systemic inflammation, and AD biomarkers. Given the expected complexity of the oral microbiome and AD-associated outcomes, this is not surprising. The identified associations provided valuable information that can be used to inform future evaluations. Finally, this was a cross-sectional study, and the findings only show correlations and do not imply a causal relationship between the oral microbiome and cognition. Further research is required, including following cognitively normal older adults longitudinally to determine if the oral microbiota may influence the development of cognitive impairment, dementia, or AD.

In summary, this study identified differences in oral microbiota composition based on cognitive function, including an increased abundance of pro-inflammatory bacteria (e.g., *Treponema*, *Parvimonas*, and *Filifactor*) and a decreased abundance of protective bacteria (*Gemella*) in those with the lowest cognitive Z Score (Q1) compared to those with higher cognitive Z Score. Whether these microbial alterations represent biomarkers of cognition and have predictive value in determining future cognitive decline remains to be determined. If an association can be positively identified, maintaining good oral hygiene or other strategies to modify the oral microbiota may help prevent cognitive decline and potentially be a new therapeutic strategy to improve cognition in patients with mild cognitive impairment or AD.

## Methods

This cross-sectional study evaluated the relationship between saliva, buccal, and lingual microbiota niches and cognition, inflammation, AD biomarkers, and brain structure.

### Study population

The current study was conducted as an ancillary study to the MIND trial, a 3-year, two-site, randomized controlled clinical trial comparing the MIND diet (Mediterranean-DASH Intervention for Neurodegenerative Delay) with a usual diet on cognitive changes among 604 older participants (68–86 years) with a family history of dementia, but without cognitive impairment, who were overweight, and consuming a suboptimal diet at the time of enrollment^58^. Of the 604 individuals at baseline, 93.4% completed 3-year cognitive assessments, and 143 adults agreed to provide saliva, buccal, and lingual samples and were included in the current study. Additionally, the MIND trial collected blood samples, assessed global cognition, and a subset of study participants underwent MRI. The study was approved by the Institutional Review Board of The Rush University Medical Center (ORA number 17042801). The study was conducted in accordance with local legislation and institutional requirements. All participants provided written informed consent to participate in the study.

### Oral microbiota collection

The participants were instructed to refrain from performing oral hygiene on the day of sample collection and to fast overnight. Samples were collected from participants with the assistance of an experienced research coordinator. The left buccal was collected by rubbing a sterile cotton-tipped applicator in circular motion over the left buccal surface 20 times, after which the swab was placed immediately into a vial containing 500 μL of sterile Zymo 1x DNA/RNA Shield solution. Using a different sterile cotton-tipped applicator, the left side of the tongue was swabbed approximately 20 times, and the swab was immediately placed in a vial containing 500 μL of sterile Zymo 1x DNA/RNA Shield solution. Each participant provided a minimum of 2 mL of unstimulated saliva by spitting directly into a 50 mL tube filled. Saliva was centrifuged, followed by aliquoting a 1:1 dilution with a sterile Zymo 2x DNA/RNA Shield solution.

### Diet

Diet was evaluated using a 142-item validated food frequency questionnaire as well as a MIND-diet screener, a 14-item measure that queried the consumption of vegetables, berries, nuts, beans, whole grains, fish, poultry, and olive oil. Higher scores on the MIND-diet screener indicated greater adherence to the MIND diet. MIND diet score was used as a covariate in the analyses.

### Blood collection and analysis

Fasting blood samples were collected by a trained phlebotomist and processed immediately after collection. AD biomarkers were measured in plasma including amyloid beta (Aβ), phosphorylated tau (pTau), neurofilament light chain (NF-L), glial fibrillary acidic protein (GFAP). Other lipid and inflammatory markers included Adiponectin, cholesterol panel, lipopolysaccharide-binding protein (LBP), Interleukin-6 (IL-6) and high sensitivity C-reactive protein (CRP). DNA was extracted to conduct APOE genotyping.

### MRI image acquisition and analysis

A subset of participants had the option to undergo a brain MRI at baseline and at the end of the study. Of the 143 individuals included in the oral microbiome evaluation, 46 elected to undergo a brain MRI. The distribution of participants with MRI data was balanced across all cognitive quartiles (Q1 = 13, Q2 = 12, Q3 = 11, Q4 = 10). MRI-derived measures of the brain were collected using a 3-Tesla Philips Achieva MRI scanner. Details of the image acquisition and processing have been described elsewhere^58^. Briefly, T1 and T2 scans were acquired, and the data from each scan underwent post-processing normalization for accurate quantification of the total brain volume, hippocampal volume, and the volume of white matter hyperintense lesions.

### Cognition

Participants completed a neuropsychological test battery of 12 performance-based tests, including assessments of executive function (Trails B, NIH toolbox flanker test), perceptual speed (Trails A, Digit Symbol Substitution Test, NIH toolbox pattern comparison test), episodic memory (immediate and delayed recall of the East Boston story and Consortium to Establish a Registry for Alzheimer’s Disease (CERAD) Word List learning, recall, and recognition), and semantic memory (category fluency, Multilingual Naming Test)^59^. The global measure is a composite of all 12 tests and is created by converting the raw scores on each test to Z Scores. For analysis, we used Z Scores as continuous measures; except for analyzing microbial diversity, we grouped the Z Scores into quartiles, with Q1 representing participants with the lowest Z Scores and Q4 representing participants with the highest Z Scores.

### Microbiome sequencing and characterization

Total DNA was extracted from the oral samples using a Maxwell RSC fecal microbiome DNA kit (Promega), with sample lysis performed using bead-beating with a TissueLyzerII device (Qiagen) and implementing ZR BashingBead Lysis tubes (Zymo). PCR was used to amplify the V4 variable region of the microbial 16S ribosomal RNA (rRNA), and the products from the PCR were then processed for amplicon sequencing, as described previously^60,61^. Sequencing was conducted using a MiniSeq sequencer with a mid-output flow cell (Illumina) at the Genomics and Microbiome Core Facility (GMCF) located at Rush University Medical Center. Raw sequences were processed through a standard bioinformatics pipeline as described previously^62^ to generate amplicon sequence variants (ASVs) and annotation tables. Negative controls were used to remove contaminants from the dataset, as described previously^62,63^. The mean number of taxa reads was calculated to quantify the sequencing depth per oral microbial niche. Among the oral niches, saliva exhibited the highest means of taxa reads (44,377.85), followed by lingual (34,625.38) and buccal (26,022.29) shown in Supplementary Table 1. Four participants were excluded from the saliva microbiome analysis because of total sequencing depths of <1000 reads.

### Statistical analysis

Data analysis was conducted using R version 4.0.3 (RRID: SCR_000432, RRID: SCR_001905). Relative microbial abundance was obtained from the read count table and converted to Phyloseq objects (RRID: SCR_013080). Using a phyloseq object, taxa were filtered based on a prevalence >10%. The data were transformed using the Centered Log Transformation “CLR” using R’s Microbiome package (RRID: SCR_024699). The alpha (α)-diversity indices were calculated using the Microbiome package (RRID: SCR_024699) on the rarefied data. Microbiome dissimilarity (beta (β)-diversity) was assessed based on the cognitive status of the Bray-Curtis dissimilarity matrix at the genus level using permutational ANOVA (PERMANOVA) with the adonis2 function of the vegan package.

Redundancy analysis was performed using the rda function of the vegan package (RRID: SCR_011950) based on the cognitive Z Score, sex, MIND diet score, education and smoking history. Subsequently, the envfit function was employed to build a model to investigate microbiome-altering factors. To investigate differential abundance, multivariable linear regression analysis was performed using the MaAslin2 package (RRID: SCR_023241) at the genus level. Uncultured genus-level taxa were suspended to the highest available taxon names. The significant features were selected based on the criteria for significance, which included a *p*-value < 0.05, and a *q*-value to control the false discovery rate of <0.25. Spearman’s rank correlation analysis was used to determine relationships between various microbes and inflammation markers, cholesterol panel, AD blood indicators, as well as MRI brain volumes and APOE genotype with Hmisc package (RRID: SCR_022497). Correlations were selected as significant based on a *p*-value < 0.05 and a *q*-value < 0.25 to control false discovery rate. Tables were created using the table1 package (RRID: SCR_024900). Graphs were generated using ggplot2 package (RRID: SCR_014601). Heatmaps were generated using the gplots package (RRID: SCR_ 025035). All data wrangling and processing was done in R before analysis using the following basic R packages: readxl (RRID: SCR_018083), dplyr (RRID: SCR_016708), tidyr (RRID: SCR_017102), and reshape2 (RRID: SCR_001905).

## Supplementary Material

Supplementary

**Supplementary information** The online version contains supplementary material available at https://doi.org/10.1038/s44400-025-00004-4.

## Figures and Tables

**Table 1 |:** Subject demographics

	Global cognitive Z score					Statistics	Post hoc
	Q1 (lowest Z score)	Q2	Q3	Q4 (highest Z score)	Total cohort		
	(*N* = 36)	(*N* = 36)	(*N* = 35)	(*N* = 36)	(*N* = 143)		
**Sex**							
Female	27 (75.0%)	23 (63.9%)	27 (77.1%)	26 (72.2%)	103 (72.0%)	*X*^2^ = 1.797, df = 3, *p* = 0.616	n/a
Male	9 (25.0%)	13 (36.1%)	8 (22.9%)	10 (27.8%)	40 (28.0%)		
**History of smoking**							
Current	1 (2.8%)	1 (2.8%)	0 (0%)	1 (2.8%)	3 (2.1%)	*X*^2^ = 5.712, df = 6, *p* = 0.456	n/a
Former	17 (47.2%)	13 (36.1%)	22 (62.9%)	18 (50.0%)	70 (49.0%)		
Never	18 (50.0%)	22 (61.1%)	13 (37.1%)	17 (47.2%)	70 (49.0%)		
**MIND diet score**							
Mean (SD)	9.43 (2.29)	8.77 (2.59)	10.4 (1.90)	9.78 (2.11)	9.59 (2.29)	ANOVA *F* = 3.18, df = 3, *p* = 0.026	Q2 v. Q3, *p* = 0.01
Median [Min, Max]	9.50 [4.50, 13.5]	8.50 [4.00, 13.5]	10.5 [6.50, 14.0]	10.0 [5.50, 13.5]	9.50 [4.00, 14.0]		
Missing	1 (2.8%)	1 (2.8%)	1 (2.9%)	0 (0%)	3 (2.1%)		
**Education**							
Technical school	10 (27.8%)	10 (27.8%)	3 (8.6%)	7 (19.4%)	30 (21.0%)	*X*^2^ = 13.459, df = 9, *p*-value = 0.143	ns
High school diploma GED	2 (5.6%)	1 (2.8%)	3 (8.6%)	0 (0%)	6 (4.2%)		
College degree	13 (36.1%)	14 (38.9%)	9 (25.7%)	11 (30.6%)	47 (32.9%)		
Post-graduate degree	11 (30.6%)	11 (30.6%)	20 (57.1%)	18 (50.0%)	60 (42.0%)		

## Data Availability

All sequencing reads generated in this study have been deposited in the National Center for Biotechnology Information (NCBI) BioProject database under accession number PRJNA1086953. Limited datasets from the MIND trial may be available for investigators upon approval of a research proposal by the MIND Trial Publication Committee.
