# Supplementary material for: Oral microbiome brain axis and cognitive performance in older adults
Source: NPJ Dement. Author manuscript; Available in PMC 2026 Mar 19. (PMC12998452; doi:10.1038/s44400-025-00004-4)
Supplement: Supplementary [file NIHMS2154447-supplement-Supplementary.pdf]

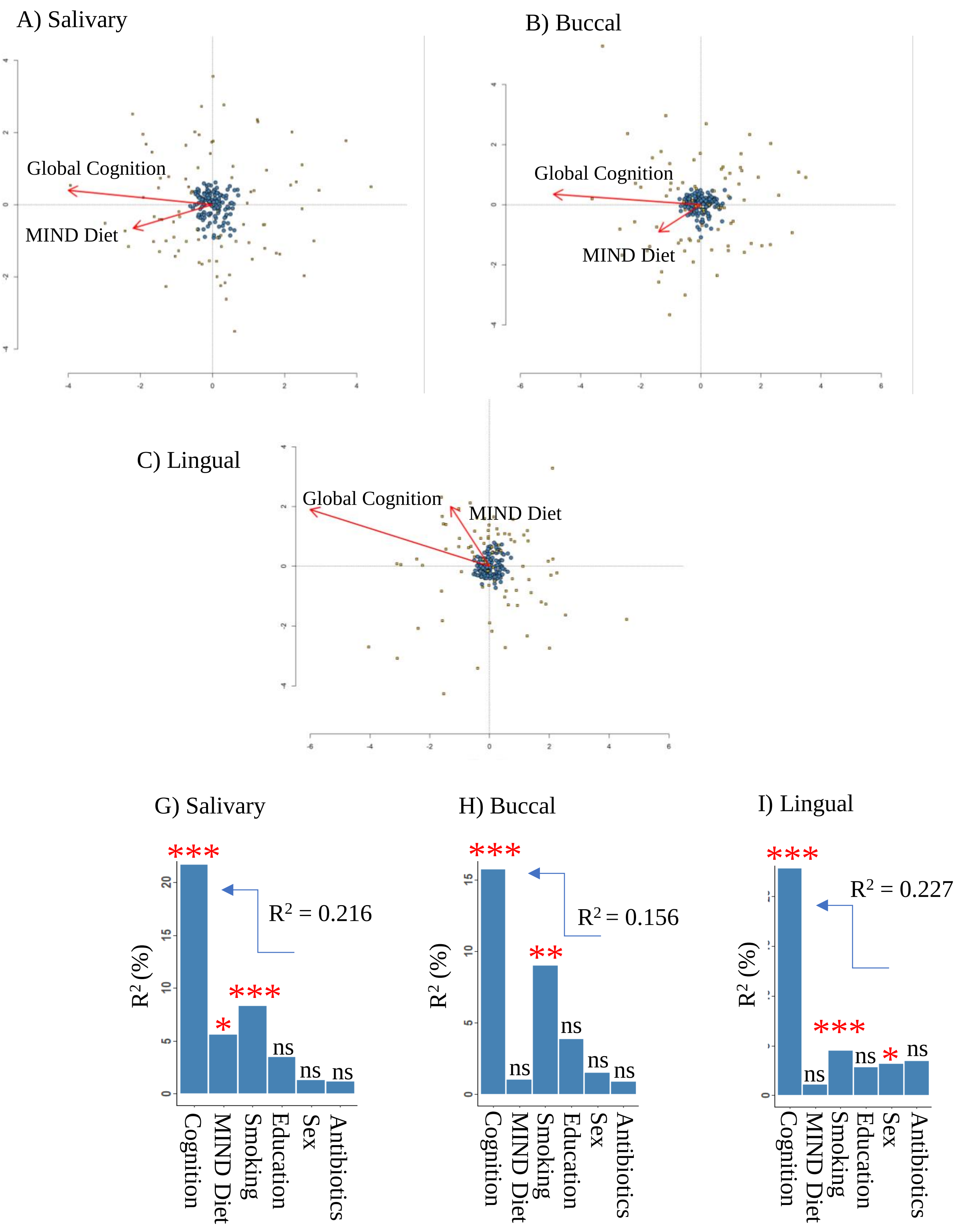

Supplementary Figure 1: RDA bi-plot; Ordination by multiple covariates showing the impact of five covariates (Global Cognition, MIND Diet Score, Smoking, Education, Sex and Oral Antibiotics) on microbiome diversity at different oral niches (A: saliva, B: buccal, and C: lingual). G. H, and I show that Cognition (Global Z) had the highest impact (Saliva: R2= 0.216, Buccal: R2= 0.156, Lingual: R2= 0.227) on microbiome diversity in all oral niches compared to MIND Diet score, smoking Education, Sex and Use of Oral Antibiotics. MIND Diet score only affected the microbiome diversity in salivary, while Sex was only significant in Lingual niche. Education and Use of Oral Antibiotics did not alter microbiome diversity in any of the oral niches.

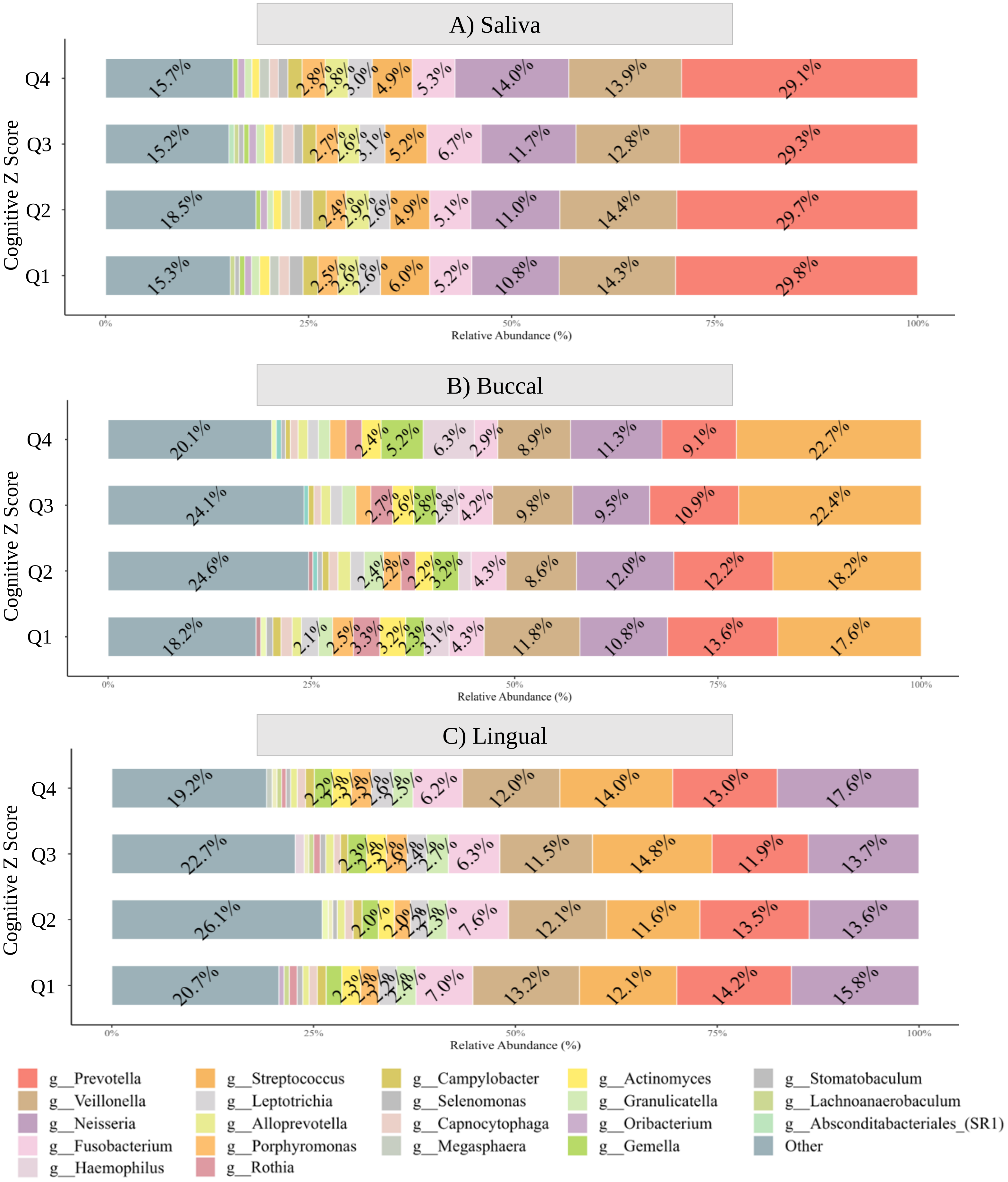

Supplementary Figure 2: Stacked bar plots show taxa at the genus level with an abundance of above 0.5% across Cognitive Z Score quartiles for each oral niche separately (A: saliva, B: buccal, and C: lingual).
Note: Text is added to bars representing taxa with an abundance above 2% to prevent overlap, but all taxa with a relative abundance higher than 0.5% are plotted.

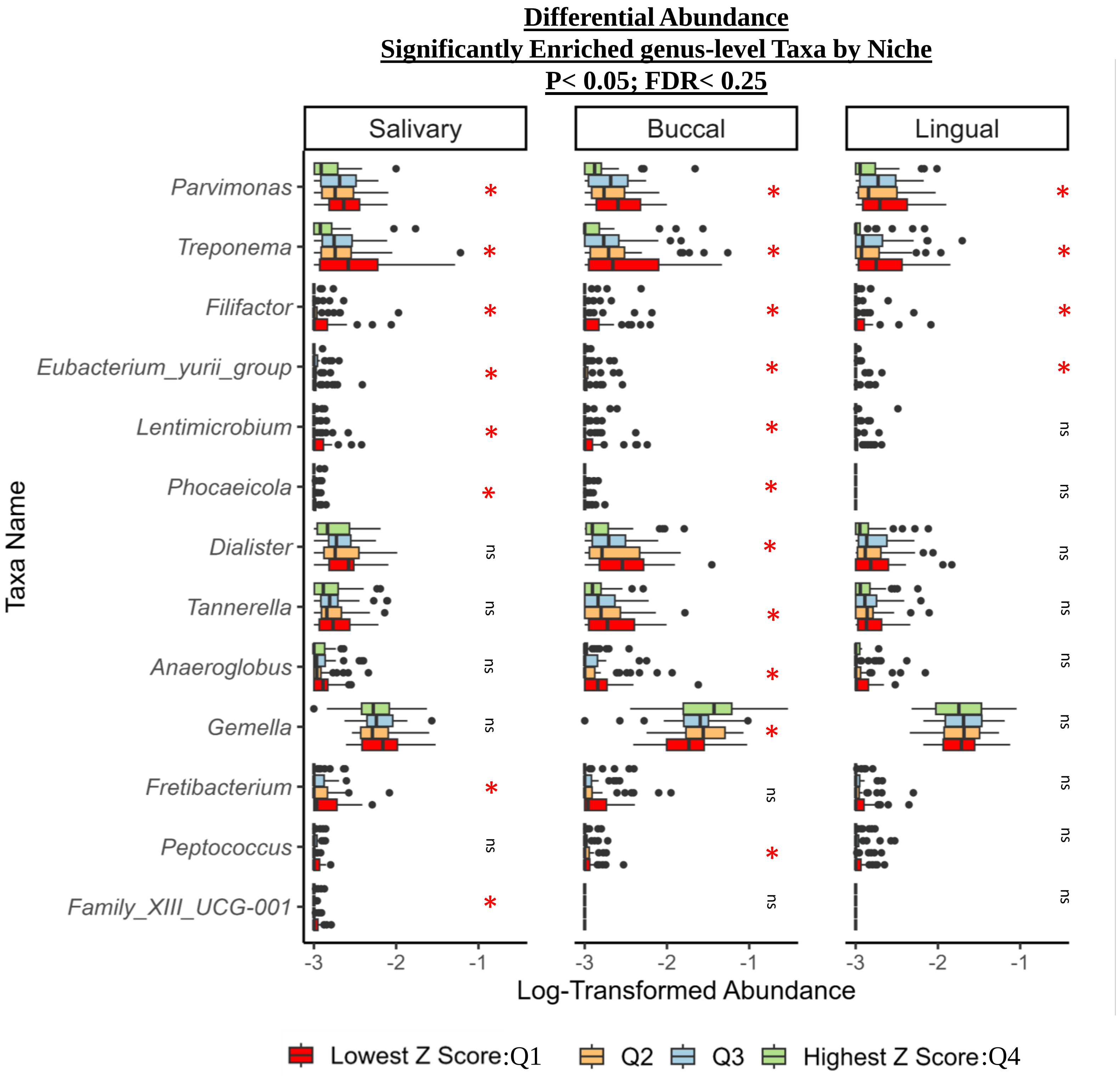

Supplementary Figure 3: **T**axa at genus level are shown on box plots based on cognitive Z Scores in each oral niche. Significant taxa are marked with a red asterisk (*). Some taxa are significantly different in all three oral niches (*Parvimonas*, *Treponema*, *Filifactor, and Eubacterium_yurii_group*), while others are significantly distinct in only one or two niches. Analysis was conducted using maaslin2 utilizing a multivariable linear model by cognitive Z Score adjusting for MIND diet, smoking history, and sex. The criteria for significance included a p-value < 0.05, q-value < 25.

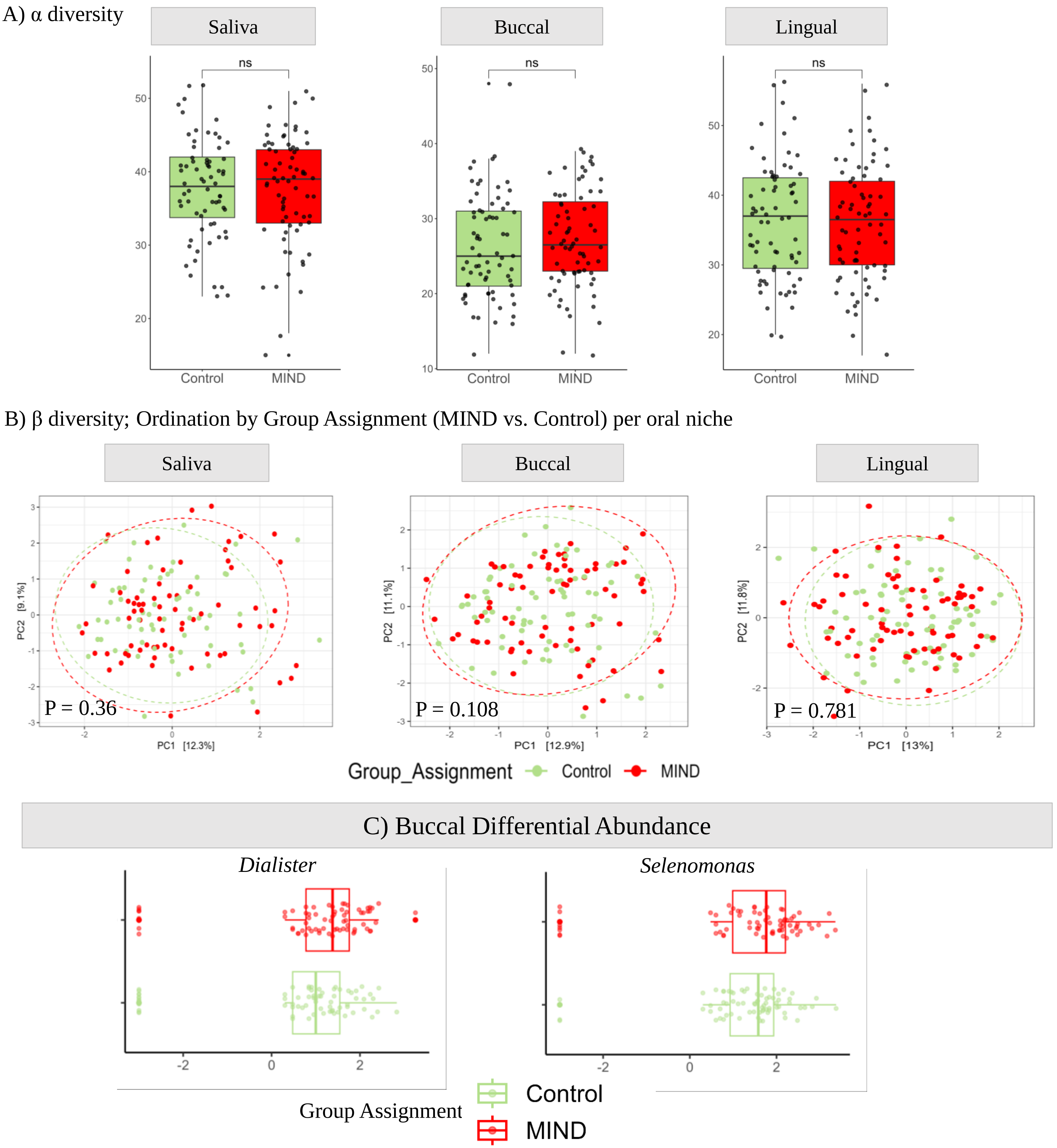

Supplementary Figure 4: Microbiome analysis by group assignment (MIND vs. Control). (A) α-diversity index of the oral microbiome community by group assignment is shown for saliva, buccal, and lingual niches. A Wilcoxon rank test showed no difference in α-diversity based on group assignment (p values: ns=not significant). (B) β-diversity by group assignment was assessed using the Bray-Curtis dissimilarity matrix and tested with PERMANOVA. Analysis revealed no statistical difference in β-diversity by group assignment ($p > 0.05$). Differential abundant analysis (univariate or multivariate while adjusting for Cognitive Z Score, Smoking and Sex) identified two taxa (*Dialister* and *Selenomonas*) that were statistically different based on group assignment ($p<0.05$, $q<0.25$).

A) α diversity

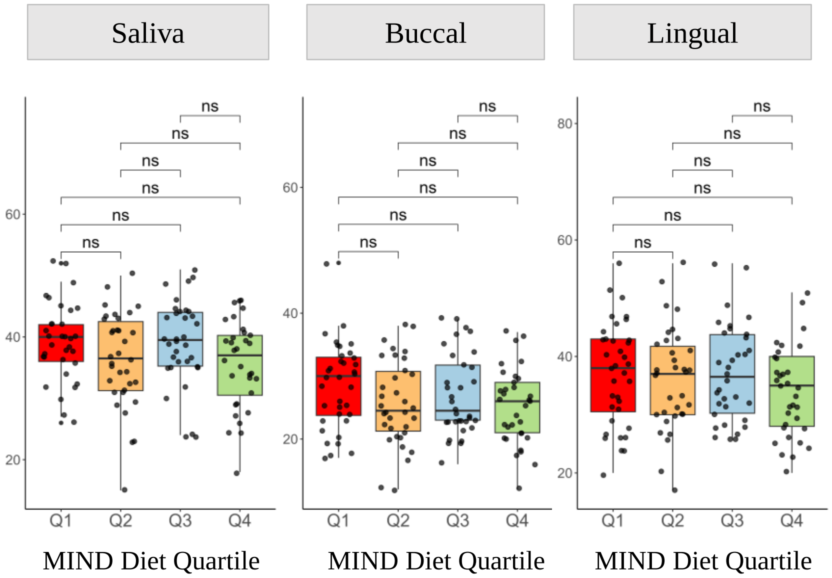

B) β diversity; PCA

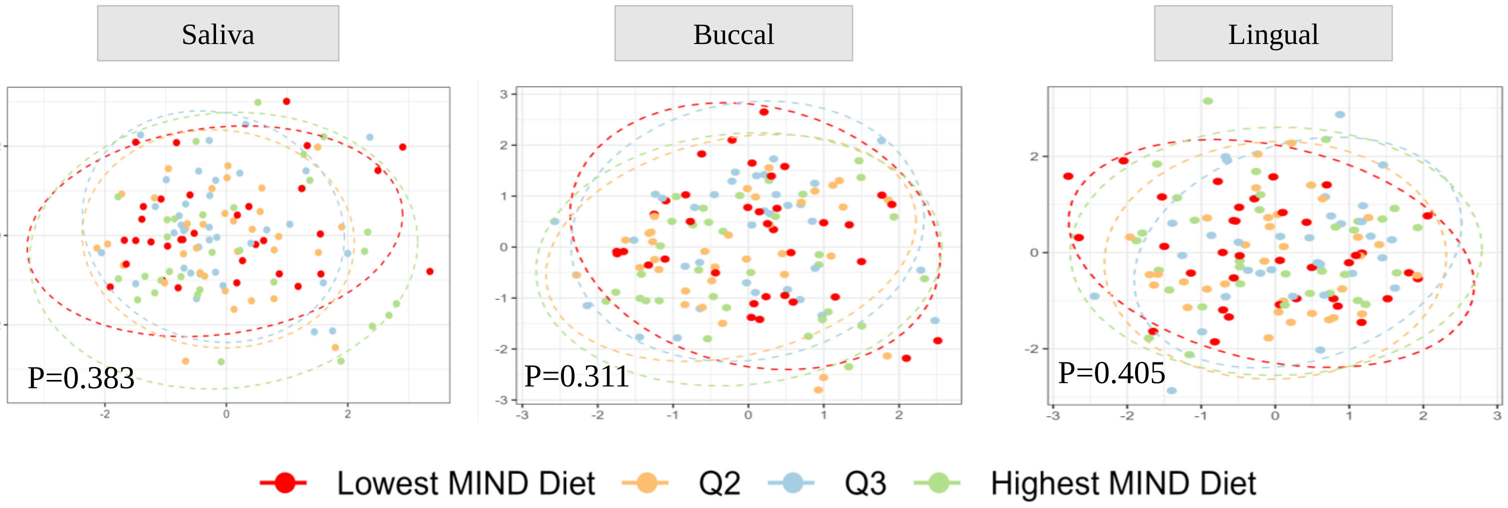

Supplementary Figure 5: Microbiome analysis by MIND Diet score regardless of group assignment. (A) α-diversity index of the oral microbiome community by MIND Diet score quartiles is shown for saliva, buccal, and lingual niches. A Wilcoxon rank test showed no difference in α-diversity based on MIND Diet Score quartiles (p values: ns=not significant). (B) Analysis of β-diversity using the Bray-Curtis dissimilarity matrix with PERMANOVA revealed no statistical difference in β-diversity across MIND Diet score quartiles ($p > 0.05$).

**Suuplementary Table 1:** Number of Taxa Reads Per Oral Niche				
Site	Number of Taxa Reads			
	Mean	SD	Median	Total_reads
Saliva	44377.85	33518.9	41595	6168521
Buccal	26022.29	12774.23	26512	3721187
Tongue	34625.38	16295.15	32107	4951430

Supplementary Table 2: Univariate analysis of various status by Global Congitive Z Score Quartiles

	Global Cognitive Z Score					
	**Q1 (Lowest Z Score)**	**Q2**	**Q3**	**Q4 (Highest Z Score)**	**Overall**	**Statistics**
	**(N=36)**	**(N=36)**	**(N=35)**	**(N=36)**	**(N=143)**	
Demographic						
**Age**						
Mean (SD)	73.8 (4.19)	73.1 (4.28)	73.3 (4.77)	71.7 (2.87)	73.0 (4.11)	ANOVA F=1.659, df=3, p=0.179
Median [Min, Max]	73.6 [68.3, 83.6]	72.0 [68.1, 86.5]	71.5 [68.2, 84.9]	71.1 [68.2, 81.3]	71.9 [68.1, 86.5]	
**BMI**						
Mean (SD)	34.1 (6.14)	33.7 (5.75)	31.8 (5.06)	32.5 (5.28)	33.1 (5.59)	ANOVA F=1.304, df=3, p=0.276
Median [Min, Max]	32.7 [25.5, 50.5]	32.6 [23.2, 51.1]	31.4 [24.9, 45.7]	31.4 [25.3, 48.3]	31.6 [23.2, 51.1]	
**Race**						
Non-White	12 (33.3%)	5 (13.9%)	3 (8.6%)	2 (5.6%)	22 (15.4%)	X2=12.891, df=3 p = 0.005
White	24 (66.7%)	31 (86.1%)	32 (91.4%)	34 (94.4%)	121 (84.6%)	
Diet						
**MIND_Diet_Score**						
Mean (SD)	9.43 (2.29)	8.77 (2.59)	10.4 (1.90)	9.78 (2.11)	9.59 (2.29)	F=3.18, df= 3, p-value = 0.026 Post-Hoc: Not significant
Median [Min, Max]	9.50 [4.50, 13.5]	8.50 [4.00, 13.5]	10.5 [6.50, 14.0]	10.0 [5.50, 13.5]	9.50 [4.00, 14.0]	
Missing	1 (2.8%)	1 (2.8%)	1 (2.9%)	0 (0%)	3 (2.1%)	
**Total_Dietary_Fiber_FFQ**						
Mean (SD)	22.6 (9.93)	22.3 (8.69)	24.5 (9.03)	24.2 (9.80)	23.4 (9.33)	F=0.503, df= 3, p-value = 0.681
Median [Min, Max]	21.5 [4.84, 46.7]	20.3 [1.52, 42.8]	26.1 [11.7, 47.4]	23.2 [7.93, 46.0]	22.0 [1.52, 47.4]	
Missing	1 (2.8%)	1 (2.8%)	0 (0%)	0 (0%)	2 (1.4%)	
**Soluble_Dietary_Fiber(FFQ)**						

Mean (SD)	6.83 (2.79)	7.02 (3.12)	7.05 (2.37)	7.22 (2.80)	7.03 (2.76)	F=0.116, df= 3, p-value = 0.951
Median [Min, Max]	6.50 [2.18, 13.3]	6.66 [0.598, 16.8]	6.97 [3.14, 14.3]	6.70 [1.64, 13.1]	6.76 [0.598, 16.8]	
Missing	1 (2.8%)	1 (2.8%)	0 (0%)	0 (0%)	2 (1.4%)	
**Insoluble_Dietary_Fiber_FFQ**						
Mean (SD)	15.7 (7.27)	15.2 (5.81)	17.4 (6.98)	17.0 (7.24)	16.3 (6.84)	F=0.795, df= 3, p-value = 0.498
Median [Min, Max]	14.4 [2.66, 33.4]	14.2 [0.928, 26.0]	18.2 [7.25, 34.7]	15.8 [4.73, 33.1]	15.3 [0.928, 34.7]	
Missing	1 (2.8%)	1 (2.8%)	0 (0%)	0 (0%)	2 (1.4%)	
Metabolic Panel						
**Hemoglobin_A1c**						
Mean (SD)	5.59 (0.486)	5.63 (0.641)	5.59 (1.12)	5.47 (0.376)	5.57 (0.705)	F=0.323, df= 3, p-value = 0.809
Median [Min, Max]	5.50 [4.70, 7.00]	5.60 [4.80, 8.50]	5.50 [1.20, 9.00]	5.50 [4.70, 6.80]	5.50 [1.20, 9.00]	
Missing	3 (8.3%)	4 (11.1%)	3 (8.6%)	3 (8.3%)	13 (9.1%)	
**Total_Cholesterol**						
Mean (SD)	187 (33.5)	181 (35.8)	182 (61.6)	191 (37.2)	185 (43.2)	F=0.379, df= 3, p-value = 0.765
Median [Min, Max]	182 [112, 248]	177 [106, 257]	196 [5.30, 276]	187 [109, 261]	187 [5.30, 276]	
Missing	3 (8.3%)	5 (13.9%)	3 (8.6%)	3 (8.3%)	14 (9.8%)	
**HDL_Cholesterol**						
Mean (SD)	59.1 (15.3)	56.2 (14.3)	69.6 (37.5)	57.2 (17.6)	60.5 (23.5)	F=2.276, df= 3, p-value = 0.083
Median [Min, Max]	56.0 [36.0, 86.0]	56.0 [34.0, 102]	59.5 [38.0, 207]	55.0 [30.0, 88.0]	56.0 [30.0, 207]	
Missing	3 (8.3%)	5 (13.9%)	3 (8.6%)	3 (8.3%)	14 (9.8%)	
**LDL_Cholesterol**						
Mean (SD)	109 (34.2)	104 (36.4)	109 (38.9)	112 (33.8)	108 (35.5)	F=0.231, df= 3, p-value = 0.875
Median [Min, Max]	105 [44.0, 187]	101 [49.0, 189]	111 [48.0, 184]	103 [54.0, 178]	105 [44.0, 189]	
Missing	3 (8.3%)	5 (13.9%)	3 (8.6%)	3 (8.3%)	14 (9.8%)	
**Oxidized_LDL**						
Mean (SD)	63.5 (20.4)	60.2 (18.4)	64.2 (22.6)	62.4 (25.7)	62.6 (21.8)	F=0.221, df= 3, p-value = 0.882
Median [Min, Max]	63.2 [24.6, 116]	56.7 [25.6, 108]	59.2 [29.7, 119]	54.4 [29.9, 134]	57.6 [24.6, 134]	
Missing	0 (0%)	2 (5.6%)	0 (0%)	0 (0%)	2 (1.4%)	
**Triglyceride**						
Mean (SD)	95.7 (30.2)	103 (43.4)	99.8 (45.3)	111 (56.1)	102 (44.6)	F=0.674, df= 3, p-value = 0.570
Median [Min, Max]	87.0 [60.0, 198]	90.0 [55.0, 271]	89.5 [45.0, 244]	91.0 [47.0, 256]	90.0 [45.0, 271]	
Missing	3 (8.3%)	5 (13.9%)	3 (8.6%)	3 (8.3%)	14 (9.8%)	

Medcations and Supplements						
**Proton Pump Inhibitors**						
No	32 (88.9%)	29 (80.6%)	26 (74.3%)	32 (88.9%)	119 (83.2%)	X-squared = 3.84, df = 3, p-value = 0.279
Yes	4 (11.1%)	7 (19.4%)	9 (25.7%)	4 (11.1%)	24 (16.8%)	
**Probiotics**						
No	32 (88.9%)	35 (97.2%)	34 (97.1%)	35 (97.2%)	136 (95.1%)	X-squared = 3.993, df = 3, p-value = 0.262
Yes	4 (11.1%)	1 (2.8%)	1 (2.9%)	1 (2.8%)	7 (4.9%)	
**NSAIDS**						
No	26 (72.2%)	29 (80.6%)	26 (74.3%)	27 (75.0%)	108 (75.5%)	X-squared = 0.740, df = 3, p-value = 0.864
Yes	10 (27.8%)	7 (19.4%)	9 (25.7%)	9 (25.0%)	35 (24.5%)	
**Aspirin**						
No	28 (77.8%)	30 (83.3%)	26 (74.3%)	28 (77.8%)	112 (78.3%)	X-squared = 0.881, df = 3, p-value = 0.83
Yes	8 (22.2%)	6 (16.7%)	9 (25.7%)	8 (22.2%)	31 (21.7%)	
History of Chronic Diseases						
**Stroke**						
No	34 (94.4%)	35 (97.2%)	33 (94.3%)	35 (97.2%)	137 (95.8%)	X-squared = 0.658, df = 3, p-value = 0.883
Yes	1 (2.8%)	1 (2.8%)	2 (5.7%)	1 (2.8%)	5 (3.5%)	
Missing	1 (2.8%)	0 (0%)	0 (0%)	0 (0%)	1 (0.7%)	
**Diabetes Mellitus**						
No	25 (69.4%)	32 (88.9%)	27 (77.1%)	32 (88.9%)	116 (81.1%)	X-squared = 8.302, df = 6, p-value = 0.217
Suspect	2 (5.6%)	1 (2.8%)	3 (8.6%)	2 (5.6%)	8 (5.6%)	
Yes	9 (25.0%)	3 (8.3%)	5 (14.3%)	2 (5.6%)	19 (13.3%)	
**High Blood Pressue**						
No	16 (44.4%)	12 (33.3%)	13 (37.1%)	20 (55.6%)	61 (42.7%)	X-squared = [illegible]

Yes	20 (55.6%)	23 (63.9%)	20 (57.1%)	15 (41.7%)	78 (54.5%)	6.207, df = 6, p-value = 0.400
Suspect	0 (0%)	1 (2.8%)	2 (5.7%)	1 (2.8%)	4 (2.8%)	
**Alcohol Use (AUDIT Score)**						
Mean (SD)	1.66 (1.57)	2.00 (1.53)	2.09 (2.30)	1.94 (1.39)	1.92 (1.71)	F=0.408, df= 3, p-value = 0.748
Median [Min, Max]	2.00 [0, 6.00]	2.00 [0, 5.00]	2.00 [0, 11.0]	2.00 [0, 4.00]	2.00 [0, 11.0]	
Missing	1 (2.8%)	0 (0%)	2 (5.7%)	1 (2.8%)	4 (2.8%)	
Work and Education						
**Education**						
Technical_school	10 (27.8%)	10 (27.8%)	3 (8.6%)	7 (19.4%)	30 (21.0%)	X-squared = 13.459, df = 9, p-value = 0.143
High_School_diploma/G	2 (5.6%)	1 (2.8%)	3 (8.6%)	0 (0%)	6 (4.2%)	
College_Degree	13 (36.1%)	14 (38.9%)	9 (25.7%)	11 (30.6%)	47 (32.9%)	
Post-graduate_degree	11 (30.6%)	11 (30.6%)	20 (57.1%)	18 (50.0%)	60 (42.0%)	
**Employment**						
Employed_full_time	5 (13.9%)	4 (11.1%)	4 (11.4%)	5 (13.9%)	18 (12.6%)	X-squared = 4.136, df = 12, p-value = 0.981
Employed_part_time	5 (13.9%)	8 (22.2%)	6 (17.1%)	7 (19.4%)	26 (18.2%)	
Retired	25 (69.4%)	23 (63.9%)	23 (65.7%)	23 (63.9%)	94 (65.7%)	
Unemployed	1 (2.8%)	1 (2.8%)	1 (2.9%)	1 (2.8%)	4 (2.8%)	
Medical_disability	0 (0%)	0 (0%)	1 (2.9%)	0 (0%)	1 (0.7%)	

Supplementary Table 3: Analysis of Alpha Diversity between all four quartiles

metrix	niche	group1	group2	p	p.signif	method	index
observed	buccal	3	4	0.032419525	*	Wilcoxon	observed
observed	buccal	3	2	0.808822612	ns	Wilcoxon	observed
observed	buccal	3	1	0.345007425	ns	Wilcoxon	observed
observed	buccal	4	2	0.087182744	ns	Wilcoxon	observed
observed	buccal	4	1	0.008962862	**	Wilcoxon	observed
observed	buccal	2	1	0.42672048	ns	Wilcoxon	observed
chao1	buccal	3	4	0.077433932	ns	Wilcoxon	chao1
chao1	buccal	3	2	0.963302154	ns	Wilcoxon	chao1
chao1	buccal	3	1	0.331084396	ns	Wilcoxon	chao1
chao1	buccal	4	2	0.097768493	ns	Wilcoxon	chao1
chao1	buccal	4	1	0.010734769	*	Wilcoxon	chao1
chao1	buccal	2	1	0.23919773	ns	Wilcoxon	chao1
evenness_s	buccal	3	4	0.413220222	ns	Wilcoxon	evenness_simpson
evenness_s	buccal	3	2	0.131028863	ns	Wilcoxon	evenness_simpson
evenness_s	buccal	3	1	0.226519221	ns	Wilcoxon	evenness_simpson
evenness_s	buccal	4	2	0.534188246	ns	Wilcoxon	evenness_simpson
evenness_s	buccal	4	1	0.699330692	ns	Wilcoxon	evenness_simpson
evenness_s	buccal	2	1	0.792522646	ns	Wilcoxon	evenness_simpson
observed	lingual	1	2	0.221344181	ns	Wilcoxon	observed
observed	lingual	1	3	0.721108967	ns	Wilcoxon	observed
observed	lingual	1	4	0.016346382	*	Wilcoxon	observed
observed	lingual	2	3	0.345173616	ns	Wilcoxon	observed
observed	lingual	2	4	0.535116765	ns	Wilcoxon	observed
observed	lingual	3	4	0.075118347	ns	Wilcoxon	observed
chao1	lingual	1	2	0.499159098	ns	Wilcoxon	chao1
chao1	lingual	1	3	0.712798837	ns	Wilcoxon	chao1
chao1	lingual	1	4	0.120036371	ns	Wilcoxon	chao1
chao1	lingual	2	3	0.52319833	ns	Wilcoxon	chao1
chao1	lingual	2	4	0.79123731	ns	Wilcoxon	chao1
chao1	lingual	3	4	0.236080306	ns	Wilcoxon	chao1
evenness_s	lingual	1	2	0.172226912	ns	Wilcoxon	evenness_simpson
evenness_s	lingual	1	3	0.196990602	ns	Wilcoxon	evenness_simpson
evenness_s	lingual	1	4	0.269082429	ns	Wilcoxon	evenness_simpson
evenness_s	lingual	2	3	0.922617493	ns	Wilcoxon	evenness_simpson
evenness_s	lingual	2	4	0.959834211	ns	Wilcoxon	evenness_simpson
evenness_s	lingual	3	4	0.98632372	ns	Wilcoxon	evenness_simpson
observed	salivary	1	2	0.165367659	ns	Wilcoxon	observed
observed	salivary	1	3	0.629318192	ns	Wilcoxon	observed
observed	salivary	1	4	0.015894097	*	Wilcoxon	observed
observed	salivary	2	3	0.33614953	ns	Wilcoxon	observed

observed	salivary	2	4	0.224889451	ns	Wilcoxon	observed
observed	salivary	3	4	0.01989365	*	Wilcoxon	observed
chao1	salivary	1	2	0.224935767	ns	Wilcoxon	chao1
chao1	salivary	1	3	0.410728311	ns	Wilcoxon	chao1
chao1	salivary	1	4	0.018454896	*	Wilcoxon	chao1
chao1	salivary	2	3	0.601567736	ns	Wilcoxon	chao1
chao1	salivary	2	4	0.190731892	ns	Wilcoxon	chao1
chao1	salivary	3	4	0.045554804	*	Wilcoxon	chao1
evenness_s	salivary	1	2	0.940787018	ns	Wilcoxon	evenness_simpson
evenness_s	salivary	1	3	0.856390283	ns	Wilcoxon	evenness_simpson
evenness_s	salivary	1	4	0.396588724	ns	Wilcoxon	evenness_simpson
evenness_s	salivary	2	3	0.928741677	ns	Wilcoxon	evenness_simpson
evenness_s	salivary	2	4	0.523163134	ns	Wilcoxon	evenness_simpson
evenness_s	salivary	3	4	0.554271434	ns	Wilcoxon	evenness_simpson

Supplementary Table 4: Univariate analysis of differentially abundance taxa by Global Z Score.

feature	niche	coef	stderr	pval	qval
g__Treponema	saliva	-0.0113604	0.003199561	0.0005273	0.0425622
g__Lentimicrobium	saliva	-0.0025459	0.00078929	0.0015722	0.0425622
g__Filifactor	saliva	-0.0040798	0.001275442	0.0017149	0.0425622
g__Family_XIII_UCG.1	saliva	-0.0013877	0.000440496	0.0020029	0.0425622
g__Eubacterium_yurii_group	saliva	-0.002024	0.000736613	0.0068104	0.0770502
g__Parvimonas	saliva	-0.0049707	0.001851579	0.0081583	0.0770502
g__Fretibacterium	saliva	-0.0035688	0.001301617	0.0069263	0.0770502
g__Phocaeicola	saliva	-0.0011278	0.000400266	0.0055554	0.0770502
g__Peptococcus	saliva	-0.0014125	0.000518797	0.0073191	0.0770502
g__Actinomyces	saliva	-0.0063273	0.002525313	0.0133969	0.0948946
c__Bacteroidia	saliva	-0.0015458	0.000610541	0.012474	0.0948946
o__Bacteroidales	saliva	-0.0012812	0.000508187	0.0128451	0.0948946
g__Defluviitaleaceae_UCG.11	saliva	-0.0013886	0.000584523	0.0189067	0.1236205
g__Dialister	saliva	-0.0042954	0.001859677	0.0223961	0.1359764
g__Mogibacterium	saliva	-0.0021949	0.001036622	0.0360349	0.204198
g__Olsenella	saliva	-0.0014677	0.000724757	0.0447975	0.2115439
g__Rikenellaceae_RC9_gut_group	saliva	-0.0012483	0.000603937	0.0406182	0.2115439
g__Eubacterium_brachy_group	saliva	-0.0014351	0.000701983	0.0428328	0.2115439
feature	niche	coef	stderr	pval	qval
g__Anaeroglobus	buccal	-0.0059519	0.001777271	0.0010408	0.0194597
g__Treponema	buccal	-0.0128249	0.003693342	0.0006851	0.0194597
g__Dialister	buccal	-0.0090539	0.002634279	0.0007735	0.0194597
g__Gemella	buccal	0.02245308	0.006582556	0.0008447	0.0194597
g__Phocaeicola	buccal	-0.00138	0.00041938	0.0012636	0.0194597
g__Filifactor	buccal	-0.003872	0.001241576	0.0022034	0.0282773
g__Peptococcus	buccal	-0.0020863	0.000705078	0.0036208	0.0333365
g__Lentimicrobium	buccal	-0.0030881	0.001026721	0.0031178	0.0333365
g__Tannerella	buccal	-0.005734	0.00195372	0.0038965	0.0333365
g__Parvimonas	buccal	-0.0059921	0.002137378	0.005768	0.0444136
g__.Eubacterium._yurii_group	buccal	-0.0018613	0.00071501	0.0102226	0.0715582
g__Eikenella	buccal	-0.00316	0.001347673	0.0204335	0.131115
g__Johnsonella	buccal	-0.0037504	0.00163199	0.0230313	0.1364164
f__Selenomonadaceae_1	buccal	-0.0048861	0.002170825	0.025944	0.1371442
g__Peptostreptococcus	buccal	-0.0034908	0.001559031	0.0267164	0.1371442
g__Fretibacterium	buccal	-0.0032154	0.001476019	0.0310356	0.1493586
g__Rikenellaceae_RC9_gut_group	buccal	-0.0014858	0.000692881	0.0337176	0.1527208
g__F0058	buccal	-0.0041832	0.002013012	0.0395137	0.1690307
feature	niche	coef	stderr	pval	qval
g__Treponema	lingual	-0.0079089	0.00225115	0.0005953	0.0482154
g__.Eubacterium._yurii_group	lingual	-0.0014229	0.000498657	0.0049758	0.1343461

g__Filifactor	lingual	-0.0028931	0.000973865	0.0034937	0.1343461
g__Parvimonas	lingual	-0.0052206	0.002112436	0.0146517	0.1887294
g__Defluviitaleaceae_UCG.011	lingual	-0.0011182	0.000437322	0.0116204	0.1887294
g__Johnsonella	lingual	-0.0032852	0.001351328	0.0163099	0.1887294
g__Lentimicrobium	lingual	-0.0016634	0.000655001	0.0121855	0.1887294
d__Bacteria	lingual	-0.0022065	0.000965015	0.0237156	0.2213955
g__Fretibacterium	lingual	-0.0021497	0.000946171	0.0245995	0.2213955
g__Anaeroglobus	lingual	-0.0024053	0.00109227	0.0292834	0.2302966
f__Prevotellaceae_1	lingual	-0.0024169	0.00116475	0.0398043	0.2302966
g__F0332	lingual	-0.0023862	0.0011148	0.0340389	0.2302966
g__.Eubacterium._brachy_group	lingual	-0.0016094	0.000764212	0.0369768	0.2302966
g__Eikenella	lingual	-0.0021809	0.001019498	0.0341413	0.2302966

Supplementary Table 5: Multivariate analysis of significantly differentially abundance taxa by cognitive Z Score adjusted by Diet, Smoking status and Sex.

feature	niche	coef	stderr	pval	qval
g__Treponema	saliva	-0.01192671	0.003372164	0.000566	0.080202
g__Family_XIII_UCG.1	saliva	-0.001555969	0.000465815	0.001099	0.116774
g__Filifactor	saliva	-0.004089956	0.00129121	0.001926	0.136409
g__Eubacterium_yurii_group	saliva	-0.00226911	0.00077652	0.004116	0.162985
g__Parvimonas	saliva	-0.005749497	0.00188356	0.002765	0.162985
g__Phocaeicola	saliva	-0.001268677	0.000421218	0.003133	0.162985
g__Lentimicrobium	saliva	-0.002392252	0.000820959	0.004218	0.162985
g__Fretibacterium	saliva	-0.003854223	0.001349495	0.005013	0.165108
feature	niche	coef	stderr	pval	qval
g__Treponema	buccal	-0.012962025	0.003757639	0.000756	0.072764
g__Gemella	buccal	0.024114463	0.006959133	0.000717	0.072764
g__Dialister	buccal	-0.009334461	0.002773463	0.001002	0.077183
g__Anaeroglobus	buccal	-0.005935345	0.001805545	0.001299	0.081184
g__Parvimonas	buccal	-0.006826287	0.002222318	0.002589	0.099682
g__Filifactor	buccal	-0.003408861	0.001197744	0.005139	0.164886
g__Phocaeicola	buccal	-0.001223419	0.000435491	0.005726	0.169591
g__Eubacterium._yurii_group	buccal	-0.001968535	0.000727159	0.007691	0.197407
g__Peptococcus	buccal	-0.001939338	0.000736398	0.009467	0.203997
g__Tannerella	buccal	-0.005277389	0.002005939	0.009538	0.203997
f__Selenomonadaceae_1	buccal	-0.005486424	0.002180433	0.01307	0.237279
g__Lentimicrobium	buccal	-0.002409216	0.000962684	0.013559	0.237279
feature	niche	coef	stderr	pval	qval
g__Treponema	lingual	-0.008539486	0.002340234	0.000379	0.080285
g__Eubacterium._yurii_group	lingual	-0.001580867	0.000522202	0.002972	0.150448
g__Filifactor	lingual	-0.002862385	0.001008351	0.005253	0.236368
g__Parvimonas	lingual	-0.006178983	0.002207326	0.005896	0.238784

Supplementary Table 6: Univariate analysis of Biomarkers by Global Cognitive Z Score Quartiles

	Global Cognitive Z Score					
	**Q1 (Lowest Z Score)** (N=36)	**Q2** (N=36)	**Q3** (N=35)	**Q4 (Highest Z Score)** (N=36)	**Overall** (N=143)	**Statistics**
Inflammatory markers						
**Total_Adiponectin**						
Mean (SD)	9.10 (6.36)	9.66 (5.36)	10.2 (6.06)	10.6 (5.51)	9.89 (5.81)	F=0.447, df= 3, p-value = 0.720
Median [Min, Max]	7.35 [1.28, 28.6]	9.52 [1.84, 21.3]	8.82 [2.62, 24.7]	10.2 [1.83, 22.0]	8.96 [1.28, 28.6]	
Missing	0 (0%)	2 (5.6%)	0 (0%)	0 (0%)	2 (1.4%)	
**Serum_LBP**						
Mean (SD)	19600 (9200)	16900 (7430)	16000 (7310)	12600 (5900)	16300 (7860)	F=5.121, df= 3, p-value = 0.00217 Post-Hoc: Q1 vs. Q4, p=0.0009 Significant
Median [Min, Max]	20200 [5850, 38300]	16800 [5850, 36900]	13200 [6180, 33300]	12100 [5850, 28500]	14200 [5850, 38300]	
Missing	1 (2.8%)	0 (0%)	0 (0%)	0 (0%)	1 (0.7%)	
**IL6**						
Mean (SD)	3.49 (2.23)	3.04 (1.93)	35.9 (152)	2.52 (1.49)	11.2 (76.4)	F=1.645, df= 3, p-value = 0.182
Median [Min, Max]	2.81 [0.848, 11.4]	2.74 [0.958, 11.0]	2.43 [0.897, 862]	2.22 [0.853, 7.19]	2.50 [0.848, 862]	
Missing	0 (0%)	2 (5.6%)	0 (0%)	0 (0%)	2 (1.4%)	
**Hs-CRP**						
Mean (SD)	5.03 (13.4)	3.12 (3.44)	3.03 (4.02)	2.22 (2.65)	3.36 (7.41)	F=0.844, df= 3, p-value = 0.472
Median [Min, Max]	2.30 [0.150, 78.6]	1.70 [0.110, 17.0]	1.55 [0.300, 20.3]	1.20 [0.200, 13.2]	1.70 [0.110, 78.6]	
Missing	3 (8.3%)	6 (16.7%)	3 (8.6%)	3 (8.3%)	15 (10.5%)	
AD Biomarkers						
**Abeta_40**						
Mean (SD)	107 (39.5)	98.0 (22.5)	102 (18.9)	101 (17.5)	102 (26.1)	F=0.657, df= 3, p-value = 0.580
Median [Min, Max]	102 [47.2, 296]	100 [4.75, 139]	99.4 [67.0, 165]	101 [64.6, 139]	100 [4.75, 296]	
Missing	0 (0%)	2 (5.6%)	1 (2.9%)	0 (0%)	3 (2.1%)	
**Abeta_42**						
Mean (SD)	7.31 (2.69)	6.95 (1.57)	6.89 (1.53)	6.91 (1.62)	7.02 (1.92)	F=0.367, df= 3, p-value = 0.777
Median [Min, Max]	6.83 [3.45, 16.3]	6.50 [3.63, 10.7]	6.99 [3.62, 10.6]	6.79 [4.22, 11.2]	6.88 [3.45, 16.3]	
Missing	0 (0%)	4 (11.1%)	1 (2.9%)	0 (0%)	5 (3.5%)	
**pTau-181**						
Mean (SD)	2.06 (1.18)	2.05 (0.875)	1.88 (0.868)	1.76 (0.735)	1.94 (0.928)	F=0.852, df= 3, p-value = 0.468
Median [Min, Max]	1.65 [0.740, 6.17]	1.79 [0.950, 4.48]	1.59 [0.740, 4.71]	1.68 [0.730, 3.31]	1.69 [0.730, 6.17]	
Missing	1 (2.8%)	3 (8.3%)	2 (5.7%)	1 (2.8%)	7 (4.9%)	

**NF-L**						
Mean (SD)	29.3 (31.0)	20.9 (7.60)	20.3 (9.28)	21.3 (14.0)	23.0 (18.4)	
Median [Min, Max]	20.8 [8.51, 193]	17.8 [11.2, 47.1]	18.6 [6.28, 48.4]	18.4 [6.76, 94.9]	18.7 [6.28, 193]	F=1.96, df= 3, p-value = 0.122
Missing	0 (0%)	2 (5.6%)	0 (0%)	0 (0%)	2 (1.4%)	
**GFAP**						
Mean (SD)	150 (71.8)	143 (60.8)	136 (48.0)	142 (66.4)	143 (62.0)	
Median [Min, Max]	132 [57.9, 378]	131 [39.9, 371]	125 [69.6, 233]	122 [54.4, 343]	126 [39.9, 378]	F=0.323, df= 3, p-value = 0.808
Missing	0 (0%)	2 (5.6%)	0 (0%)	0 (0%)	2 (1.4%)	
Brain Volume (MRI)						
**Total Hippocampal volume**						
	n=13	n=12	n=11	n=10	n=46	
Mean (SD)	6980 (451)	7570 (893)	7200 (823)	7500 (591)	7300 (727)	F=1.79, df= 3, p-value = 0.164
Median [Min, Max]	6930 [6460, 7950]	7520 [5620, 8860]	7140 [5860, 8720]	7660 [6260, 8290]	7220 [5620, 8860]	
Missing	23 (63.9%)	24 (66.7%)	24 (68.6%)	26 (72.2%)	97 (67.8%)	
**White Matter (wm) volume**						
Mean (SD)	372000 (37500)	397000 (72500)	371000 (37700)	397000 (28200)	384000 (47900)	
Median [Min, Max]	376000 [306000, 426000]	387000 [271000, 547000]	360000 [325000, 413000]	397000 [358000, 445000]	385000 [271000, 547000]	F=1.04, df= 3, p-value = 0.387
Missing	23 (63.9%)	24 (66.7%)	24 (68.6%)	26 (72.2%)	97 (67.8%)	
**Gray Matter (gm) volume**						
Mean (SD)	555000 (40200)	579000 (58200)	577000 (68100)	580000 (44500)	572000 (53000)	
Median [Min, Max]	546000 [496000, 612000]	579000 [444000, 669000]	586000 [459000, 690000]	573000 [527000, 659000]	574000 [444000, 690000]	F=0.64, df= 3, p-value = 0.593
Missing	23 (63.9%)	24 (66.7%)	24 (68.6%)	26 (72.2%)	97 (67.8%)	
**Intracrania (icv) volume**						
Mean (SD)	1370000 (116000)	1440000 (197000)	450000 (18100(	1450000 (121000)	1430000 (157000)	
Median [Min, Max]	1370000 [1160000, 1550000]	1410000 [1060000, 1790000]	1410000 [1230000, 1860000]	1440000 [1310000, 1730000]	1400000 [1060000, 1860000]	F=0.715, df= 3, p-value = 0.549
Missing	23 (63.9%)	24 (66.7%)	24 (68.6%)	26 (72.2%)	97 (67.8%)	
APOE4 Status						
**APOE4_Identifier**						
Heterozygote	12 (33.3%)	12 (33.3%)	10 (28.6%)	7 (19.4%)	41 (28.7%)	
Homozygote	1 (2.8%)	0 (0%)	0 (0%)	1 (2.8%)	2 (1.4%)	X-squared = 4.158, df = 6, p-value = 0.655
Non_Carrier	23 (63.9%)	24 (66.7%)	25 (71.4%)	28 (77.8%)	100 (69.9%)	

**Supplementary Table 7:** Univariate analysis of Oral Microbiota by Blood AD Biomarkers						
feature	niche	biomarker	coef	stderr	pval	qval
g__Rothia	Salivary	Abeta_40	0.02101	0.00315	6.1807E-10	5.2536E-08
g__Rothia	Salivary	Abeta_42	0.01343	0.0035	0.00019273	0.01638222
g__Alloprevotella	Salivary	NF.L	-0.01281	0.0053	0.01708648	0.1815439
g__Gemella	Salivary	NF.L	0.00628	0.00243	0.01069733	0.15154557
g__Lachnoanaerobaculum	Salivary	NF.L	-0.00624	0.00209	0.00343586	0.08368331
g__Megasphaera	Salivary	NF.L	-0.00943	0.00382	0.01489895	0.18091577
g__Mogibacterium	Salivary	NF.L	-0.00236	0.00105	0.02538397	0.23973753
g__Oribacterium	Salivary	NF.L	-0.00776	0.00264	0.00393804	0.08368331
g__Rothia	Salivary	NF.L	0.02408	0.00296	2.3381E-13	1.9874E-11
g__Stomatobaculum	Salivary	NF.L	-0.00738	0.00272	0.00750783	0.12763315
g__Streptococcus	Salivary	NF.L	0.01895	0.00638	0.00354009	0.08368331
g__Rothia	Salivary	pTau.181	0.01536	0.00348	2.1624E-05	0.00183801
feature	niche	biomarker	coef	stderr	pval	qval
g__Rothia	Lingual	Abeta_40	0.02928	0.0059	1.9802E-06	0.0001604
g__Stomatobaculum	Lingual	Abeta_40	-0.00908	0.00325	0.00595128	0.24102689
g__Abiotrophia	Lingual	Abeta_42	0.00837	0.00285	0.00385807	0.107639
g__Granulicatella	Lingual	Abeta_42	-0.01663	0.00568	0.00398663	0.107639
g__Rothia	Lingual	Abeta_42	0.02394	0.00615	0.00015526	0.01257596
g__Stomatobaculum	Lingual	Abeta_42	-0.00922	0.00329	0.00579729	0.11739517
g__Lentimicrobium	Lingual	GFAP	0.00201	0.00066	0.00260447	0.21096218
g__Rothia	Lingual	NF.L	0.02485	0.00608	7.4158E-05	0.00600678
